# Combined Photoredox Catalysis for Value-Added Conversion of Contaminants at Spatially Separated Dual Active Sites

**DOI:** 10.34133/research.0055

**Published:** 2023-02-21

**Authors:** Jieyuan Li, Ruimin Chen, Kaiwen Wang, Yan Yang, Jielin Wang, Weiping Yang, Shengyao Wang, Guidong Yang, Fan Dong

**Affiliations:** ^1^ Research Center for Carbon-Neutral Environmental and Energy Technology, Institute of Fundamental and Frontier Sciences, University of Electronic Science and Technology of China, Chengdu 611731, China.; ^2^ Beijing Key Lab of Microstructure and Properties of Advanced Materials, Beijing University of Technology, Beijing 100124, China.; ^3^ School of Environmental Science and Engineering, Institute of Environmental Health and Pollution Control, Guangdong University of Technology, Guangzhou 510006, Guangdong, China.; ^4^ College of Science, Huazhong Agricultural University, Wuhan 430070, China.; ^5^ XJTU-Oxford Joint International Research Laboratory of Catalysis, School of Chemical Engineering and Technology, Xi’an Jiaotong University, Xi’an 710049, China.

## Abstract

As 2 indispensable counterparts in one catalysis system, the independent reduction and oxidation reactions require synergetic regulation for cooperatively promoting redox efficiency. Despite the current success in promoting the catalytic efficiency of half reduction or oxidation reactions, the lack of redox integration leads to low energy efficiency and unsatisfied catalytic performance. Here, we exploit an emerging photoredox catalysis system by combining the reactions of nitrate reduction for ammonia synthesis and formaldehyde oxidation for formic acid production, in which superior photoredox efficiency is achieved on the spatially separated dual active sites of Ba single atoms and Ti^3+^. High catalytic redox rates are accomplished for respective ammonia synthesis (31.99 ± 0.79 mmol g_cat_^−1^ h^−1^) and formic acid production (54.11 ± 1.12 mmol g_cat_^−1^ h^−1^), reaching a photoredox apparent quantum efficiency of 10.3%. Then, the critical roles of the spatially separated dual active sites are revealed, where Ba single atoms as the oxidation site using h^+^ and Ti^3+^ as the reduction site using e^−^ are identified, respectively. The efficient photoredox conversion of contaminants is accomplished with environmental importance and competitive economic value. This study also represents a new opportunity to upgrade the conventional half photocatalysis into the complete paradigm for sustainable solar energy utilization.

## Introduction

Independent reduction and oxidation reactions are 2 indispensable counterparts in ubiquitous catalytic systems, which proceed simultaneously to use the electrons (e^−^) and holes (h^+^) generated by catalysts, respectively [1–4]. The adequate enhancement of combined redox capacity is imperative to maximize the utilization efficiency of input energy and achieve optimum catalytic performance. In this regard, heterogeneous photocatalysis can be developed as an ideal platform to attain the synergetic utilization of e^−^–h^+^ pairs for e^−^-driven reduction and h^+^-driven oxidation reactions [5–8]. However, individual reductive or oxidative pathways (half reactions) are separately developed in most research works [9–11], which largely overlooks the advantage of redox integration in general photocatalysis reactions. To establish a complete photoredox system, the charge proximity between the catalytic sites for e^−^ and h^+^ requires precise control to restrain the disordered charge transfer and undesired charge recombination [12–14]. Hence, the spatially separated active sites should be coherently designed for a delicate balance involving reduction and oxidation reactions, which could achieve a complete photoredox system for synergistically promoted catalytic reactions [11,15,16].

The e^−^ or h^+^ sacrificial agents have been widely applied in photocatalysis systems, in which the e^−^/h^+^ is consumed rapidly to facilitate the corresponding half reaction [17,18]. Despite the current success in promoting the half catalytic efficiency, the unfavorable energy loss and extra consumption of sacrificial agents appeal to researchers to exploit alternative strategies for full utilization of photocatalysis reactions at both ends. Some combined photoredox attempts have been made recently to address this pivotal issue, such as the H_2_O_2_ reduction-assisted methane oxidation [19], contaminant oxidation-assisted hydrogen evolution [20,21], and water oxidation-assisted nitrate reduction [6]. Unfortunately, these routes are still limited in the domain of using pseudo-sacrificial agents, given that the products from the sacrificial half reaction do not have high economic value. Furthermore, it should be noted that the photocatalytic overall water splitting route is a pioneer to combine the reduction and oxidation photocatalysis, primarily through depositing reductive and oxidative cocatalysts onto substrates to catalyze respective hydrogen and oxygen evolution reactions. However, these cocatalysts are almost reaction-specific, impeding their extension to general reaction systems [22–24]. Besides, inspired by the blueprint of electrocatalysis, it arises that the independent reduction and oxidation reactions could proceed at physically separated electrochemical cells with directed charge transfer [2,25,26], in which superior redox efficiency is thereby expected. Based on these valuable explorations, it is encouraging that the combined photoredox catalysis is highly feasible, provided that the spatially separated dual active sites can be accurately constructed for the respective reduction and oxidation reactions, where both can be utilized for value-added transformations.

**Fig. 1.**
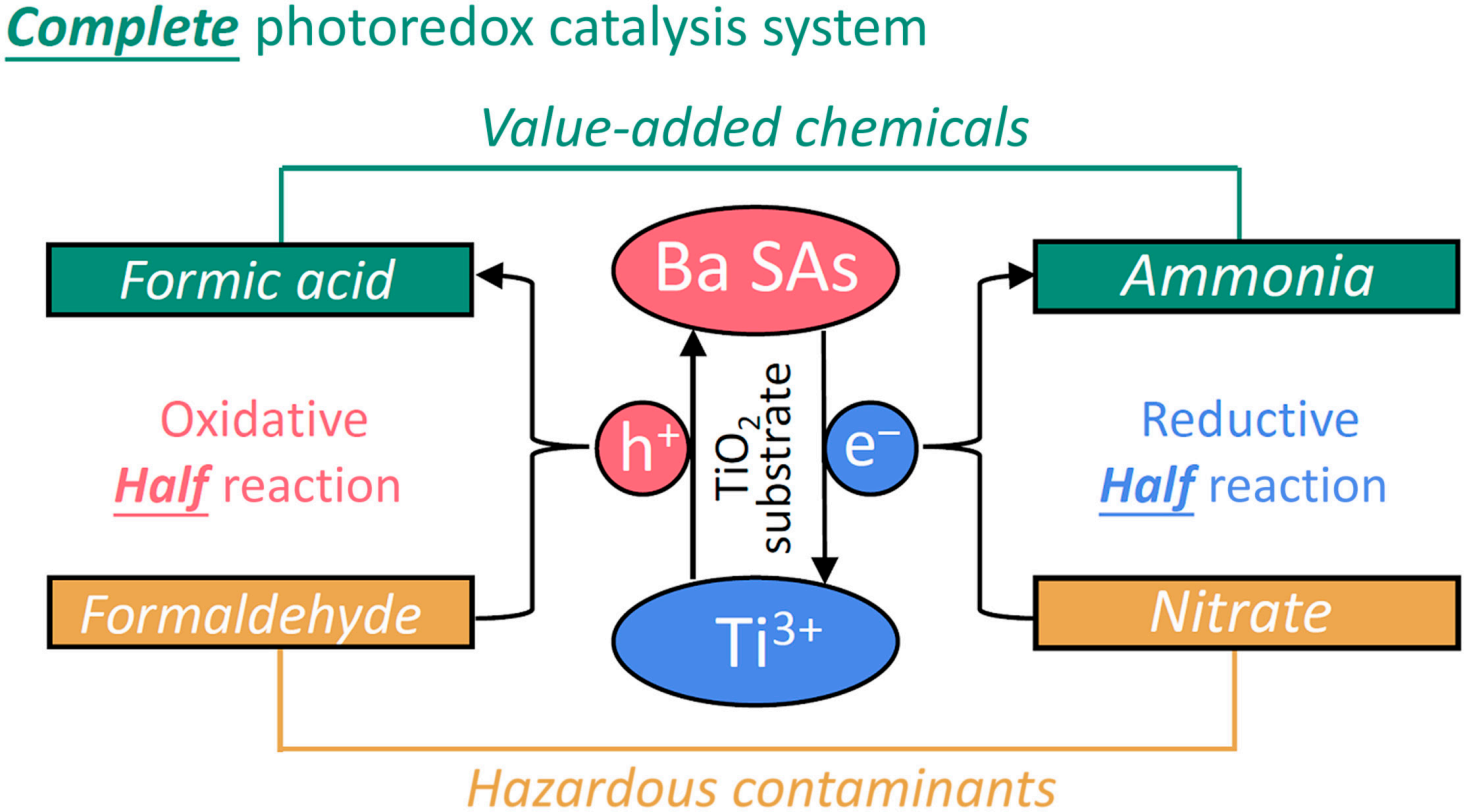
Illustration for the photoredox catalysis system.

Here, we provide an emerging and elegant photoredox catalysis route by synergetic nitrate reduction for ammonia synthesis and formaldehyde oxidation for formic acid (FA) production. A cocatalyst-free photoredox catalyst is designed via a facile method by depositing Ba single atoms (Ba SAs) onto Ti^3+^-enriched TiO_2_ nanosheets with oxygen vacancies (Ba-TNS_OV_). The charge proximity between the spatially separated dual active sites is precisely regulated, where the Ba SAs for the oxidation site and Ti^3+^ for the reduction site are constructed, respectively. As major pollutants in wastewater and atmosphere [27–31], the value-added conversion of contaminative nitrate and formaldehyde is synchronously achieved on Ba-TNS_OV_, in which a photoredox apparent quantum efficiency of 10.3% is reached. The design innovation of this complete photoredox catalysis system is illustrated in Fig. 1. A strong dependency of the catalytic efficiency on Ba content and Ti^3+^ concentration is observed. The subtle roles of the dual active sites and the detailed reaction mechanism are unraveled. By the provision of contaminants and solar light as the only feedstocks, the value-added conversion with complete photoredox reactions is accomplished with superior catalytic efficiency and economic value. This study represents a general strategy to upgrade the conventional half photocatalysis reaction into the complete paradigm for sustainable energy and environmental applications.

## Results and Discussion

### Synthesis of Ba-TNS_OV_ photoredox catalysts

**Fig. 2.**
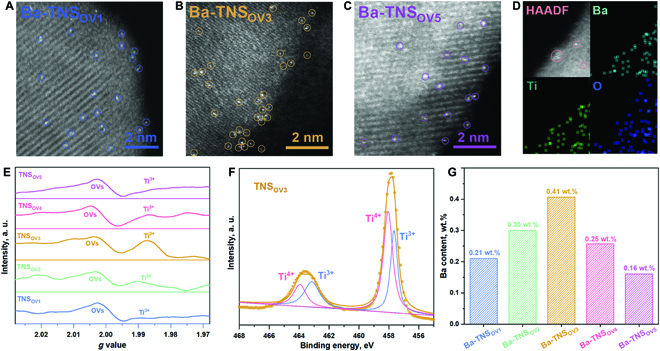
Catalyst fabrication and characterization (A to G). HAADF-STEM images for Ba SAs on TNS with different Ti^3+^ concentrations (A to C). HAADF-STEM image and corresponding elemental mappings of Ba, Ti, and O elements in Ba-TNS_OV3_ (D). Low-temperature (140 K) solid EPR results (E). XPS spectra for TNS_OV3_ (F). ICP measurements for Ba content determination (G).

The TNS samples with oxygen vacancies (OVs) are synthesized with the solvothermal method by using different solvents to achieve the regulation of corresponding Ti^3+^ concentration (labeled as TNS_OV1_ to TNS_OV5_), in which the Ba^2+^ is operando added into the solvothermal precursor to construct the Ba SAs (labeled as Ba-TNS_OV1_ to Ba-TNS_OV5_). The powder x-ray diffraction (XRD), scanning electron microscopy (SEM), and transmission electron microscopy (TEM) results (Figs. S1 to S3) reveal that the crystal structure and morphology of nanosheet-like TiO_2_ are well maintained after Ti^3+^ inducement and Ba SAs deposition. Moreover, it is identified by high-angle annular dark-field scanning transmission electron microscopy (HAADF-STEM) observation that the Ba SAs are uniformly distributed on the TNS surfaces (Fig. 2A to C) without other types of Ba species noted (Fig. S4), which indicates the selective construction of Ba SAs on TNS surface. Besides, the HAADF-STEM elemental mappings (Fig. 2D) confirm that the deposited SAs are composed of Ba element.

Then, the formation of OV-induced Ti^3+^ is clarified by applying the solid electron paramagnetic resonance (EPR) technology. As depicted in Fig. S5, no obvious signals are detected at room temperature. In order to improve the test accuracy, the low-temperature (140 K) EPR measurements are conducted (Fig. 2E). It is found that the almost equal intensity of OV signals (*g* = 2.000) are detected under low temperature (140 K) for all the samples [32,33], in which the intrinsic defects in TNS are determined as the primary source of OVs. Importantly, it is worth noting that the solvent-induced Ti^3+^ generation is obviously viewed (*g* = 1.987) [34,35]. The distribution of Ti^3+^ intensity is confirmed from TNS_OV1_ to TNS_OV5_, in which the maximum value is achieved on TNS_OV3_. The formation of Ti^3+^ and deposition of Ba SAs are also revealed by x-ray photoelectron spectroscopy (XPS) results (Fig. 2F and Figs. S6 to S8) [36,37]. Interestingly, as identified by the inductive coupled plasma (ICP) results, the evolution pattern of Ba content (Fig. 2G) is in accordance with that of the Ti^3+^ concentration (Fig. 2E). The Ba content is increased along the Ti^3+^ concentration, which identifies that the optimum amount of Ba SAs and Ti^3+^ dual active sites are constructed on Ba-TNS_OV3_ compared to that of the other catalysts.

### Identification of spatially separated dual photoredox active sites

**Fig. 3.**
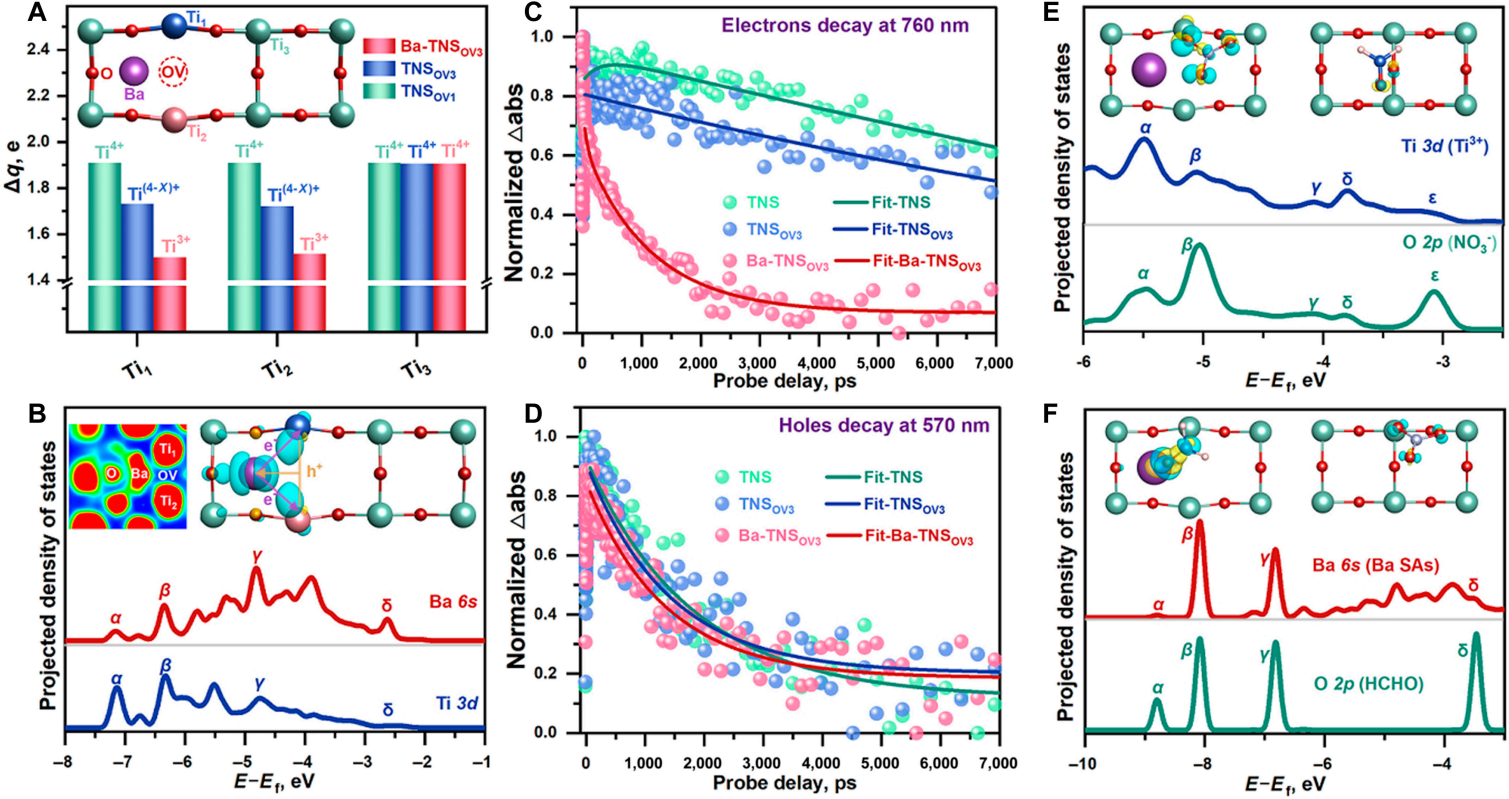
Dual active sites identification (A to D). Calculated Bader effective charge (A) and optimized microstructure (A inset). Calculated PDOS (B), electronic localization function (B inset), and charge difference distribution (B inset); the isosurface is set to 0.001 eV Å^−3^; charge accumulation is marked in blue and charge depletion is marked in yellow. Kinetics of electrons (C) and holes (D) in TA spectra under the probe wavelengths of 760 and 570 nm, respectively [51,52]. Calculated PDOS (E) and charge difference distribution (E inset) for NO_3_^−^ activation. Calculated PDOS (F) and charge difference distribution (F inset) for HCHO activation. The isosurface for (E inset) and (F inset) is set to 0.003 eV Å^−3^; charge accumulation is marked in blue and charge depletion is marked in yellow.

After the fabrication of TNS_OV_ and Ba-TNS_OV_ photoredox catalysts, it is essential to identify the structure of dual active sites. As depicted in the inset of Fig. 3A and Fig. S9, the structural disorder is observed after the formation of OVs and Ba SAs, respectively, as indicated by the density functional theory (DFT) calculation results, leading to local charge redistribution. It is first confirmed that the inducement of OVs drives the neighboring Ti_1_ and Ti_2_ atoms from Ti^4+^ (Δ*q* = 1.90 e) to Ti^(4−*X*)+^ (Δ*q* = 1.73 e), which is in accordance with the XPS results (Fig. 2F). Moreover, the further reduction of Ti^(4−*X*)+^ is achieved by the construction of Ba SAs to generate Ti^3+^ (Δ*q* = 1.49 e). The electron acquirement of Ti_1_ and Ti_2_ atoms is increased in the order of TNS_OV1_ < TNS_OV3_ < Ba-TNS_OV3_. In contrast, the irrelevant Ti_3_ atom remains electronically stable (Δ*q* = 1.90 e), which, in turn, certifies that Ti^3+^ generation is contributed by both OV and Ba SA construction. Due to the spatial separation of Ba SAs and Ti^3+^ in this local region, it is concluded that the dual photoredox active sites are produced.

To unravel the respective roles of Ba SAs and Ti^3+^ active sites in the photoredox reaction, a comprehensive electronic structure analysis is then performed. As suggested by the projected density of states (PDOS) results (Fig. 3B), the overlap of energy level for Ba *6s* electrons with that of neighboring Ti *3d* is noted, illustrating the formation of the covalent bond between Ba and Ti atoms via the directional charge transfer within these atoms. Then, it is observed by the electronic localization function and charge difference distribution (Fig. 3B, inset) that these covalent bonds dominantly originate from the electron depletion from Ba to the corresponding Ti atoms during the deposition of Ba SAs. That is, owing to the charge redistribution at this Ba-Ti local region, the directed electron transfer channel is constructed from Ba to Ti atoms. Under light excitation, the e^−^-enriched Ti^3+^ can serve as the reduction active site, which should deliver extra e^−^ to the reductive reactant during the photoredox reaction. Meanwhile, the h^+^-enriched nature of Ba SAs leads to the formation of oxidation active sites. After the establishment of separated dual active sites of Ba SAs and Ti^3+^, the appealing regulation of proximity between oxidation and reduction active sites is realized, which also promotes the light utilization (Fig. S10).

Most importantly, transient absorption (TA) measurement is applied to further investigate the charge transfer kinetics within the dual photoredox active sites. As shown in Fig. 3C, the lifetime of electrons is decreased in the order of TNS_OV1_ < TNS_OV3_ < Ba-TNS_OV3_. This result confirms that Ba SAs act as the electron sink to trap the light-generated electrons. Subsequently, rapid electron transfer can be realized along the channel of Ba SAs → Ti^3+^ to participate in the reductive reaction. On the contrary, the elevated decay rate of holes (Fig. 3D) is observed by introducing the Ti^3+^ sites (1,405 ps) compared to that of the pristine TNS (1,808 ps), which indicates the reversed hole transfer from Ti^3+^ to Ba SAs in comparison with that of the electron transfer. The combined DFT calculation and TA results indicate a directional charge transfer and an efficient charge separation at the dual active sites of Ba SAs and Ti^3+^. It is expected that superior photoredox catalytic efficiency can be accomplished on these Ba-TNS_OV_ catalysts. In addition, the NO_3_^−^ (Fig. 3E) and HCHO (Fig. 3F) adsorption properties are calculated to further reveal the interfacial e^−^/h^+^ transfer between redox active sites and reactants. As shown in Fig. 3E, intense charge exchange between Ti^3+^ and NO_3_^−^ (O atom) is noted in the calculated PDOS results for the NO_3_^−^@Ba-TNS_OV_ composite. The directed electron delivery from Ti^3+^ to O atom promotes the electron transfer from Ti^3+^ to NO_3_^−^ (Fig. 3E, inset), which again testifies the Ti^3+^ as reduction active sites. Accordingly, the hole transfer from Ba SAs to HCHO (O atom, Fig. 3F) induces the subsequent HCHO oxidation reaction, further identifying the Ba SAs as oxidation active sites.

### Tests of ammonia synthesis efficiency

**Fig. 4.**
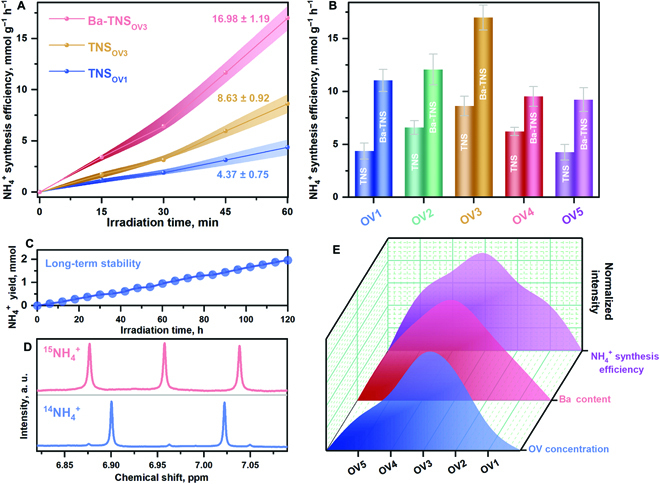
Ammonia synthesis efficiency evaluation (A to E). Ammonia synthesis efficiency promoted by Ti^3+^ and Ba SA inducement (A). Ammonia synthesis efficiency comparison within all the synthesized catalysts (B). Isotope-labeled ^1^H NMR results for ^14^NH_4_^+^ and ^15^NH_4_^+^ synthesis (C). Long-term stability test (D). Normalized volcanic curves of NH_4_^+^ synthesis efficiency, Ba content, and Ti^3+^ concentration (E). In order to provide suitable concentration values for accurate detection, different standard curves for NH_4_^+^ detection using ion chromatography (IC) are applied for the test in (A) and (B) (Fig. S11, 0.4 to 2.0 ppm) and (C) (Fig. S12, 1 to 20 ppm), respectively. The error bars were determined based on the calculated standard error of 3 parallel tests. In the ammonia synthesis efficiency test (A and B), 1.0 mg of the as-synthesis catalyst sample was dispersed in 100 ml of reaction solution containing 100.0 mg l^−1^ of NO_3_^−^-N and 10.0 ml of ethylene glycol solution. In the isotype tracking experiment (C), 10 mg l^−1^ of ^14^NO_3_^−^ and ^15^NO_3_^−^ was used as the feedstock, respectively, while the other test parameter was the same as that of the ammonia synthesis efficiency test. As for the long-term stability test (D), the concentration of NO_3_^−^-N was elevated to 500.0 mg l^−1^ to ensure enough feedstock, and the catalyst dosage was set to 50.0 mg.

The efficiency test toward nitrate reduction for ammonia synthesis is first conducted to verify the advantage of the as-fabricated TNS_OV_ and Ba-TNS_OV_ catalysts. As shown in Fig. 4A and B and Fig. S13, the catalytic efficiency of the TNS substrate is observably enhanced by Ti^3+^ inducement, in which the optimum value is reached at TNS_OV3_ (8.63 ± 0.92 mmol g^−1^ h^−1^ for ammonia synthesis). After Ba SA deposition, the further enhanced charge separation capacity endows the Ba-TNS_OV_ samples with elevated activity in comparison with that of the corresponding TNS_OV_. Specifically, the ammonia synthesis efficiency is promoted in the order of TNS_OV1_ (4.37 ± 0.75 mmol g_cat_^−1^ h^−1^) < TNS_OV3_ (8.63 ± 0.92 mmol g_cat_^−1^ h^−1^) < Ba-TNS_OV3_ (16.98 ± 1.19 mmol g_cat_^−1^ h^−1^), confirming the distinct advantages of Ti^3+^ inducement and Ba SA deposition, respectively. The balance of nitrogen species is checked, which indicates that the selectivity of NH_4_^+^ is as high as 95.02% from NO_3_^−^ reduction on Ba-TNS_OV3_ (Fig. S14). Besides, the nitrogen source of nitrate reduction for ammonia synthesis is clarified by using ^14^NO_3_^−^ and ^15^NO_3_^−^ solutions as the respective reactants for isotope-labeled measurement. As illustrated in the ^1^H NMR results (Fig. 4C), the corresponding ^14^NH_4_^+^ and ^15^NH_4_^+^ anions are detected, confirming that the generated ammonia is directly derived from nitrate reduction and the potential contribution of the other contaminative N-containing species can be ignored. In addition, long-term tests are performed to determine the stability of Ba-TNS_OV_ catalysts (Fig. 4D). The initial NO_3_^−^ concentration (500.0 mg l^−1^) and catalyst dosage (50.0 mg) are increased for long-term tests, in order to guarantee sufficient feedstock in the 120 h of photoredox reaction. It is found that the stable production of ammonia is realized, yielding a total amount of 1.96 mmol of ammonia within 120 h. After the long-term tests, the catalyst structure is also well maintained (Figs. S15 to S18).

After the coherent evaluation of Ba-TNS_OV_ catalysts toward nitrate reduction for ammonia synthesis, it is observed that the volcanic curves of ammonia synthesis efficiency with Ba content and Ti^3+^ concentration are consistently established (Fig. 4E). Hence, the direct contribution of Ti^3+^ inducement and Ba SA construction to the elevated catalytic efficiency is remarkably noted.

### Development of the complete photoredox system

In the above section, the combined nitrate reduction (NO_3_^−^RR) reaction and ethylene glycol oxidation reaction (EG-OR) is developed as a prototype, aiming at verifying the catalyst performance and evaluating the feasibility of the photoredox reaction system [38]. It is apparent that the EG-OR is not economically competitive as the oxidation route, and the corresponding NO_3_^−^RR performance is currently not satisfied when combined with the EG-OR. Hence, it requires developing another cooperative oxidation reaction, which not only facilitates the nitrate to ammonia synthesis efficiency but also demonstrates importance for sustainable environmental and energy applications. Since the compromise of reduction and oxidation reaction rates is important to conduct a complete photoredox route, to accomplish a cooperative and efficient photoredox system with high economic value and environmental implications, we choose the synergetic NO_3_^−^RR and formaldehyde oxidation reaction (HCHO-OR), which works as a complete photoredox paradigm for the synthesis of both ammonia and FA from the value-added conversion of contaminative nitrate and formaldehyde. Also, the strong reducibility of HCHO should endow the corresponding NO_3_^−^RR with enhanced reaction efficiency.

**Fig. 5.**
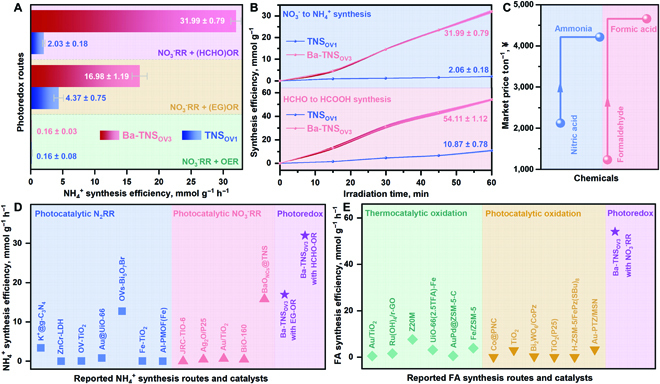
Complete photoredox catalytic route development (A to E). Ammonia synthesis efficiency comparison between different photoredox routes (A). Complete photoredox efficiency evaluation in the combined NO_3_^−^ reduction and HCHO oxidation route (B). The market price of the photoredox reactants and products (C). Comparison of NH_4_^+^ synthesis rate using photoredox catalysis with different reported routes and catalysts (D). Comparison of FA production rate using photoredox catalysis with different reported routes and catalysts (E). The standard curve for FA detection using IC is provided in Fig. S19. The error bars were determined based on the calculated standard error of 3 parallel tests.

**Fig. 6.**
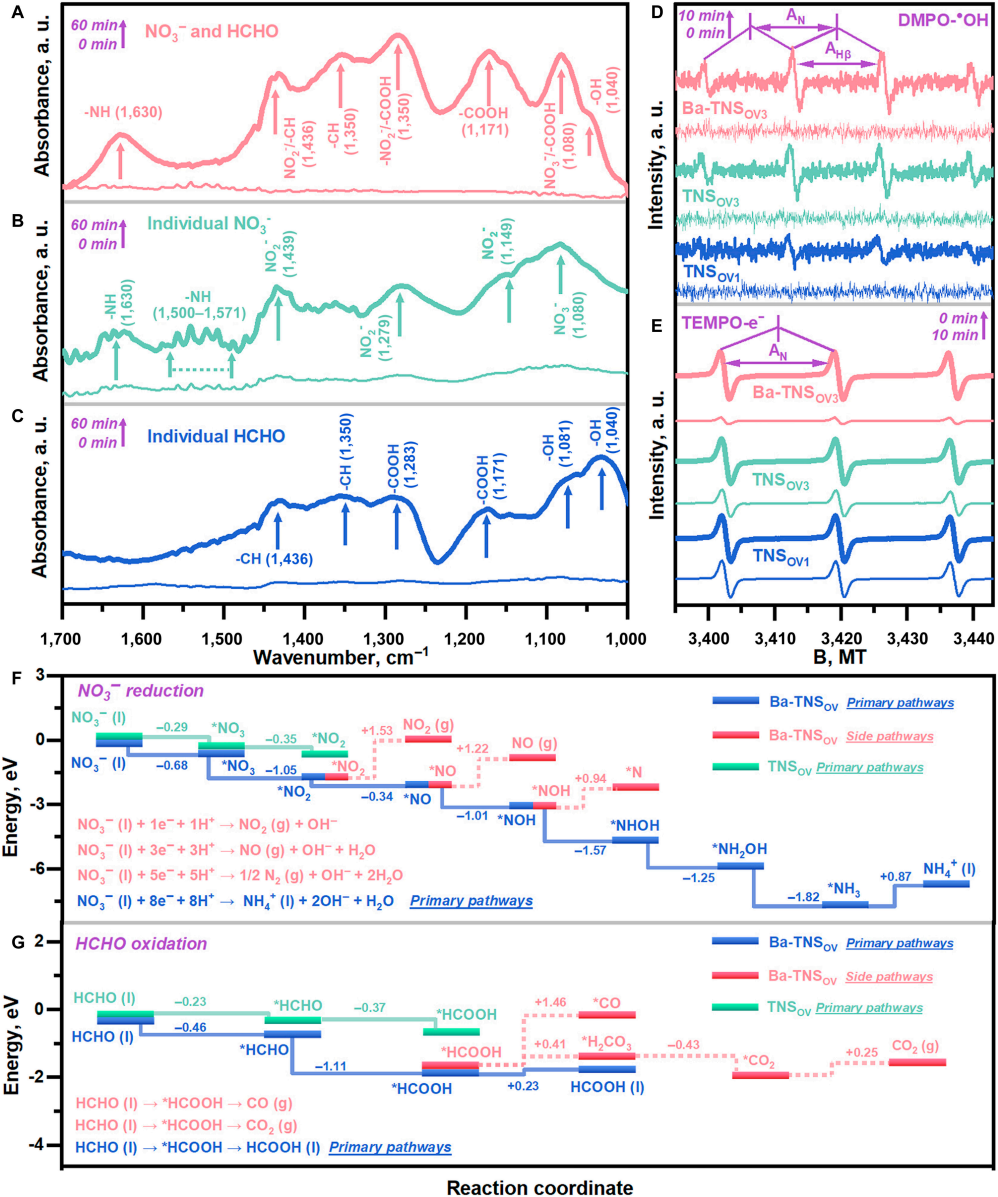
Photoredox reaction mechanism investigation (A to E). In situ DRIFTS spectra for synchronous NO_3_^−^ reduction and HCHO oxidation (A), individual NO_3_^−^ reduction (B), and HCHO oxidation (C) under light irradiation; the range of the *y*-axis is set identical for (A) to (C). Liquid EPR results for active radicals’ detection of oxidative DMPO-^●^OH (D) and reductive TEMPO-e (E) generation during the photoredox reaction. Calculated Gibbs free energy diagram for NO_3_^−^ reduction (F) and HCHO oxidation (G) reaction mechanisms.

As presented in Fig. 5A, the ammonia synthesis rate on Ba-TNS_OV3_ is obviously promoted by the cooperation of NO_3_^−^RR and HCHO-OR (31.99 ± 0.79 mmol g^−1^ h^−1^) in comparison with that of the route of NO_3_^−^RR coupling with EG-OR (16.98 ± 1.19 mmol g^−1^ h^−1^), which directly certifies the advancement of tailored photoredox routes. Meanwhile, the inferior activities are observed in the system of combined NO_3_^−^RR and oxygen evolving reaction (Fig. S19). Hence, it is informed that the independent reduction and oxidation reactions are tunable and their matching is essential for the synergetic photoredox system. Diverse impacts are expected by combining different redox counterparts for specific performance targets of energy conversion and environmental remediation. Most importantly, it is found that abundant FA is produced on Ba-TNS_OV3_ along with the superior ammonia synthesis, which exceeds that of the TNS_OV1_ (Fig. 5B). The respective generation rates for ammonia and FA are determined to be as high as 7.80 mol g_Ba_^−1^ h^−1^ (31.99 ± 0.79 mmol g_cat_^−1^ h^−1^) and 13.19 mol g_Ba_^−1^ h^−1^ (54.11 ± 1.12 mmol g_cat_^−1^ h^−1^) on Ba-TNS_OV3_. The apparent quantum efficiency for photoredox reaction of combined NO_3_^−^RR and HCHO-OR is calculated to be 10.3%. Besides, the charge-related reaction rates are calculated to be 108.22 mmol h^+^ g^−1^ h^−1^ for HCHO-HCOOH oxidation (2 h^+^) and 255.92 mmol e^−^ g^−1^ h^−1^ for NO_3_^−^-NH_4_^+^ reduction (8 e^−^). It is true that the redox efficiencies are not stoichiometrically balanced, in which the excess h^+^ should be consumed for the overoxidation of HCHO into carbonate or CO_2_. Some related evidences for HCHO overoxidation were also provided in the diffused reflectance infrared Fourier transform spectroscopy (DRIFTS) and DFT results (Fig. 6).

Benefitting from the successful redox cooperation of combined NO_3_^−^-NH_4_^+^ synthesis and HCHO-FA production, the feasible photoredox system is accomplished toward the value-added conversion of contaminants, given the contaminative nature of reactants at both ends and increased economic value of redox products (Fig. 5C). In this photoredox system, ammonia and FA's respective production rates are superior in the related fields (Fig. 5D and E and Tables S1 and S2). The ammonia synthesis efficiency also exceeds that of our previous work under the same reaction condition [38].

### Photoredox reaction mechanism identification

To obtain the mechanistic insight into the photoredox system, the liquid–solid system of in situ DRIFTS is firstly designed to dynamically monitor the reaction process of corresponding NO_3_^−^RR and HCHO-OR. As shown in Fig. 6A, the characteristic peaks regarding NO_3_^−^ (1,080 cm^−1^) and -CH (1,436 and 1,350 cm^−1^) are obviously detected as the synchronous NO_3_^−^RR and HCHO-OR that proceed under light irradiation, which is reasonable since the liquid DRIFTS system contains enough NO_3_^−^ and HCHO reactants, leading to extra signals for their adsorption during the whole test process. Notably, the accumulation of NO_2_^−^ (1,436 and 1,350 cm^−1^) and NH_4_^+^ (1,630 cm^−1^) is observed as the reductive products [39–41]. Similarly, the HCHO-OR is identified with an increase in -COOH (1,350 and 1,171 cm^−1^) and -OH (1,080 and 1,040 cm^−1^) species [42–45]. As comparison, the in situ DRIFTS for individual NO_3_^−^RR (Fig. 6B) and HCHO-OR (Fig. 6C) is conducted. It is found that the corresponding characteristic peaks are in accordance with that of the synchronous NO_3_^−^RR and HCHO-OR (Fig. 6A). However, the production of NH_4_^+^ (1,630 cm^−1^ in Fig. 6B) and formate (1,283 and 1,171 cm^−1^ in Fig. 6C) is obviously restrained, which directly indicates that the construction of complete photoredox system is beneficial for respective reduction and oxidation reactions. The complete in situ DRIFTS spectra are also provided in Figs. S21 to S23. Then, the active radicals, which are responsible for catalyzing the respective oxidation and reduction reactions, are unraveled. As shown in Fig. 6D and Fig. S24, the generation of 5,5-dimethyl-1-pyrroline-*N*-oxide (DMPO)-^●^OH (3,395, 3,410, 3,424, and 3,439 G) is facilitated in the order of TNS_OV1_ < TNS_OV3_ < Ba-TNS_OV3_, which agrees with the pattern of catalytic efficiency. Similarly, the consumption of 4-oxo-2,2,6,6-tetramethyl-1-piperidinyloxy-oxyl (TEMPO; 3,402, 3,419, and 3,435 G) is also defined along the path of TNS_OV1_ < TNS_OV3_ < Ba-TNS_OV3_ (Fig. 6E and Fig. S25). Since the consumption of TEMPO is directly contributed by the light-generated e^−^, the improvement of Ti^3+^ and Ba SAs construction on the e^−^–h^+^ separation and transfer properties is further verified. It is deduced that the ^●^OH and e^−^ are the corresponding active species for oxidation and reduction reactions in this photoredox catalysis. The corresponding hyperfine-coupling constants of DMPO-^●^OH (A_Hβ_ = A_N_ = 14.3 G) and TEMPO (A_N_ = 17.6 G) are identified, respectively.

The detailed dynamic behavior of NO_3_^−^RR and HCHO-OR is subsequently revealed by DFT calculations to support the experimental results. As depicted in Fig. 6F and Figs. S26 to S28, the adsorption and activation of NO_3_^−^ on Ba-TNS_OV_ (−0.68 eV) are remarkably enhanced in comparison with that of TNS (−0.29 eV), which accelerates the subsequent NO_3_^−^RR. Then, the enhancement of *NO_3_ to *NO_2_ reduction is found on Ba-TNS_OV_ with the reaction energy of −1.05 eV, lower than that of the pristine TNS (−0.35 eV), which confirms the advantage of Ti^3+^-reductive site construction. Moreover, due to the multiple reaction routes with electrons for NO_3_^−^RR, 4 competing reduction pathways are comprehensively investigated (1e^−^-, 3e^−^-, 5e^−^-, and 8e^−^-induced reduction), which generate NO_2_, NO, N_2_, and NH_4_^+^ as the reductive products, respectively. By the energy criterion at each division point, the primary reduction reaction pathways are identified as the NO_3_^−^ to NH_4_^+^ synthesis (blue line in Fig. 6F), obviously exceeding that of the 3 competitive side reactions (red lines in Fig. 6F). It is certified that the potential by-product generation of intermediated NO_2_, NO, and N_2_ is impeded to guarantee selective ammonia synthesis. The calculation results for HCHO-OR (Fig. 6G) also confirm the advantage of the Ba SAs-oxidative site in comparison with that of the TNS, which guarantees the selective oxidation of HCHO molecule into HCOOH rather than the production of CO and CO_2_ as side products. By applying the closely combined in situ technology and atomic-level calculations, the complete photoredox reaction mechanism is established in the combined NO_3_^−^RR and HCHO-OR photoredox route.

## Conclusions

In this study, an emerging and elegant photoredox route is developed by the cooperation of combined nitrate reduction for ammonia synthesis and formaldehyde oxidation for FA production, where the photogenerated holes and electrons can be fully utilized. Superior photoredox efficiency is accomplished by the construction of a Ba-TNS_OV_ photoredox catalyst. The critical roles of the dual active sites are unraveled, which determines the Ba SAs as oxidation active sites and Ti^3+^ as reduction active sites, respectively. The precisely regulated charge proximity between the spatially separated dual active sites leads to efficient charge separation and reactant conversion. The detailed photoredox reaction mechanisms of selective nitrate reduction and formaldehyde oxidation on Ba-TNS_OV_ have been unraveled on the basis of coherent in situ characterizations and theoretical calculations. The value-added conversion of contaminants is accomplished in this tunable photoredox system, which demonstrates the great potential for working as a multifunctional technology for both environmental remediation and energy conversion. The presented photoredox catalysis scheme could provide numerous opportunities to upgrade the conventional half photocatalysis reaction into the complete paradigm for sustainable solar energy utilization.

## Materials and Methods

### Chemicals

All chemicals were used as received without further treatment.

### Catalyst fabrication

The synthesis of the TNS substrate was conducted by modification of the reported work [46,47]. In a typical fabrication process, 3.5 ml of HF solution was added dropwise into 25 ml of Ti (OBu)_4_•(TBOT) under magnetic stirring. After intensive mixing, a gel-like solution was formed. Then, the mixed solution was transferred to a Teflon autoclave (50 ml) and heated at 180°C for 24 h. After cooling down to room temperature, the obtained powder was washed with ethanol (EtOH) and distilled water (DI) at least 10 times, respectively, and collected by centrifugation at 10,000 rpm for 5 min. Finally, the obtained powder was dried at 80°C overnight.

As for the synthesis of TNS_OV1-OV5_, a re-solvothermal method was applied to construct Ti^3+^. In a typical synthesis procedure, 0.1 g of the as-fabricated TNS sample was added to 60 ml of the specific solutions (OV1: 100% of DI, OV2: 50% of DI and 50% of EtOH, OV3: 100% of EtOH, OV4: 50% of EtOH and 50% of EG, and OV5: 100% of EG). After enough magnetic stirring, the mixture was transferred to a Teflon autoclave (100 ml) and heated at 150°C for 18 h. After naturally cooling down to room temperature, the obtained samples were collected by washing with EtOH and DI 3 times, respectively. After that, the TNS_OV1-OV5_ samples were dried at 80°C overnight. The synthesis of Ba-TNS_OV1-OV5_ catalysts followed the same procedure as TNS_OV1-OV5_ by introducing an extra 0.01 g of BaCl_2_·2H_2_O into the mixture of TNS substrate and specific solutions.

### Characterization

The crystal information was investigated by the XRD technology (Shimadzu XRD-6100). The morphology was examined by SEM (FEG ESEM XL30) and TEM (FEI Talos F200S). The formation of Ba SAs was verified by the HAADF-STEM (JEOL JEM-ARM200F) with spherical aberration correction to observe the atomic morphology. The XPS (Thermo Scientific K-Alpha+) was used to verify the chemical structure. The solid-state EPR (Bruker EMXnano) was utilized to identify the OV signals at the temperature of 140 K. The light absorption properties were examined by a UV–Vis (ultraviolet–visible) spectrophotometer (Shimadzu UV-2450). Time-dependent fluorescence emission decay spectra (PicoQuant Fluo-time 300) were recorded to determine the lifetime of the light-generated carriers. The active radicals for catalyzing the photoredox reaction were identified by the liquid EPR (Bruker EMXnano), in which the DMPO and TEMPO were used as the trapping agents to confirm the generation of DMPO-^●^OH and TEMPO-e^−^ in DI dispersion.

The component of the Ba element was investigated by ICP (Agilent ICPOES730). The experiment was conducted as follows: 0.1 g of catalyst sample was placed in a Teflon tube (50 ml) for decomposition. Concentrated nitric acid (5 ml) was then added into the tube. The sealed sample was transferred into a stainless reaction still for decomposition at 190°C for 10 h. After naturally cooling down to room temperature. The obtained solution was transferred into a volumetric flask for a constant volume of 25 ml by DI. Known mass and volume were inputted into the ICP-OES equipment for detection. The concentration was quantified by the corresponding standard substances.

### TA measurement

The output pulse of a 1-kHz Ti (the sapphire regenerated amplifier: Solstice, Spectra-Physics) was set into 2 separated beams. One of the beams was applied as the excitation light source of an optical parametric amplifier (OPA, TOPAS prime, NIR-UV-Vis. LIGHT CONVERSION Inc.). The light source was set to 340 nm for excitation and was cut off at 500 Hz by an optical chopper. The other beam was used as an excitation light source of another OPA. The output of OPA was used as the wavelength-tunable probe and referred light source (500 to 2,600 nm). Si and InGaAs photodetectors were applied to detect the probe and referred light depending on the probe light wavelength (500 to 1,000 nm and 1,000 to 2,600 nm, respectively).

### Calculation details

The DFT calculations were performed with the software of the Vienna ab initio simulation package (VASP 5.4.1) by using the Perdew–Burke–Ernzerhof exchange-correlation functional [48–50]. The cutoff energy was limited to 400 eV. K points were set to 5 × 5 × 1 for both dynamic and electronic optimization. Structure relaxation was reached after the residual forces were smaller than 0.01 eV Å^−1^. The width of Gaussian smearing was set to 0.2 eV. The initial calculation model was provided in Fig. S8.

The adsorption energy (*E*_ads_) and desorption energy (*E*_des_) were calculated as follows, respectively:

$$E_{\mathrm{ads}}=E_{\mathrm{tot}}-E_{\text{surf}}-E_{\mathrm{mol}}$$ (1)

$$E_{\mathrm{des}}=-E_{\mathrm{ads}}$$ (2)

where *E*_tot_, *E*_surf_, and *E*_mol_ refer to the calculated energy of the adsorption complex, the catalyst support, and the pristine molecules, respectively.

The Gibbs free energy variation (Δ*G*) between the initial state (IS) and final state (FS) was calculated as follows:

$$\Delta G=E_{\mathrm{FS}}-E_{\text{IS}}+\Delta E_{\mathrm{ZPE}}-T\Delta S$$ (3)

where *E*_FS_, *E*_IS_, Δ*E*_ZPE_, *T*, and Δ*S* depict the calculated DFT energy for FS and IS, the variation of zero-point energy, room temperature (298.15 K), and entropy, respectively.

### Photoredox efficiency test

One milligram of as-synthesis catalyst sample was dispersed in 100 ml of reaction solution containing 100.0 mg l^−1^ of NO_3_^−^-N and 10.0 ml of EG or HCHO solution. Then, the reaction mixture was transferred into a photocatalysis reactor (Merry Change MC-GF250). The operation temperature was controlled at 25°C by a circulating chiller. After degassing with Ar at 50 ml min^−1^ for 30 min under continuous magnetic stirring, a 300-W Xe lamp (Merry Change MC-X301B) was applied at the light source. The NO_3_^−^, NO_2_^−^, NH_4_^+^, and HCOOH were detected by ion chromatography (Shimadzu IC-16). As for the long-term stability experiment, the concentration of NO_3_^−^-N was elevated to 500.0 mg l^−1^ to ensure enough feedstock. The catalyst dosage was set to 50.0 mg. After the long-term test, the remaining catalyst was collected and washed for characterization. The ^15^N isotope labeling measurement was performed by using 10 mg l^−1^ of K^15^NO_3_ and K^14^NO_3_ as the feedstock, respectively. The generated ^15^NH_4_^+^ and ^14^NH_4_^+^ were tested by the ^1^H NMR (Bruker 400M).

### Apparent quantum yield calculation

The quantum efficiency is defined by the ratio of the effective electrons and holes used for product formation to the total input photon flux. The reduction of NO_3_^−^ to NH_4_^+^ requires 8 electrons, and the oxidation of HCHO to HCOOH requires 2 holes.

$$\begin{array}{c} \mathrm{QE}\%=\frac{\text{Effective electrons}+\text{holes}}{\text{Total photons}}\times \\ 100\%=\left(8X+2Y\right)N/\Theta \mathrm{TS}\times 100\% \end{array}$$ (4)

where *X* refers to the yields of NH_4_^+^ and *Y* refers to the yields of HCOOH. *N* depicts the Avogadro’s number, Θ is the photon flux, *T* is the irradiation time, and *S* is the illumination area.

The following example is calculated based on the photoredox efficiency from combined NO_3_^−^RR and HCHO-OR (Fig. 5A and B): *X* = 3.2 × 10^−5^ mol, *Y* = 5.4 × 10^−5^ mol, *N* = 6.022 × 10^23^ mol^−1^, Θ = 1.97 × 10^17^ s^−1^ cm^−2^, *T* = 3,600 s, and *S* = 3.0 cm^2^.

QE% = (8 × 3.2 × 10^−5^ mol + 2 × 5.4 × 10^−5^ mol)/(1.97 × 10^17^ s^−1^ cm^−2^ × 3,600 s × 3.0 cm^2^) = 10.3%.

### In situ DRIFTS investigation

In situ DRIFTS (Bruker INVENIO R) measurement was conducted to monitor the photoredox reaction process, in which an in situ reaction cell (Harrick) and a reaction chamber was equipped. Before measurement, the catalyst was dispersed in 100 mg l^−1^ of NO_3_^−^-N solution, 10 vol.% of EG solution, and the mixture of 100 mg l^−1^ of NO_3_^−^-N and 10 vol.% of EG, respectively. Highly purified He was continuously purged into the reaction system to acquire the inert atmosphere. A point light source of Xe lamp (Bobei BBZM-1) was used as the light source. The IR signals were continuously detected during the adsorption process in the dark and the reaction process with the light on.
