# Supplementary material for: Combined Photoredox Catalysis for Value-Added Conversion of Contaminants at Spatially Separated Dual Active Sites
Source: Research (Wash D C). 2023 Feb 21;6:0055. doi: 10.34133/research.0055 (PMC10076036; doi:10.34133/research.0055)
Supplement: Supplementary Materials — Figs. S1 to S28 Tables S1 and S2 [file research.0055.f1.docx]

***Supporting Information for***

**Combined Photoredox Catalysis for Value-Added Conversion of Contaminants at Spatially Separated Dual Active Sites**

**Photoredox Conversion of Contaminants into Chemicals**

Jieyuan Li^1+^, Ruimin Chen^1+^, Kaiwen Wang^2^, Yan Yang^3^, Jielin Wang^1^, Weiping Yang^1^, Shengyao Wang^4^, Guidong Yang^5^ and Fan Dong^1^*

1 Research Center for Carbon-Neutral Environmental & Energy Technology, Institute of Fundamental and Frontier Sciences, University of Electronic Science and Technology of China, Chengdu 611731, China.

2 Beijing Key Lab of Microstructure and Properties of Advanced Materials, Beijing University of Technology, Beijing 100124, China.

3 School of Environmental Science and Engineering, Institute of Environmental Health and Pollution Control, Guangdong University of Technology, Guangzhou, 510006, Guangdong, China.

4 College of Science, Huazhong Agricultural University, Wuhan 430070, China.

5 XJTU-Oxford Joint International Research Laboratory of Catalysis, School of Chemical Engineering and Technology, Xi’an Jiaotong University, Xi’an 710049, China.

*Correspondence should be addressed to Fan Dong: [dfctbu@126.com](mailto:dfctbu@126.com), [dongfan@uestc.edu.cn](mailto:dongfan@uestc.edu.cn).

+ These authors contributed equally to this work.

Table of Context

**Supplementary Figures3**

Figure S1 XRD patterns3

Figure S2 SEM images4

Figure S3 TEM images5

Figure S4 HAADF-STEM images6

Figure S5 EPR results at room temperature7

Figure S6 High-resolved XPS spectra for O 1*s*8

Figure S7 High-resolved XPS spectra for Ti 2*p* 9

Figure S8 High-resolved XPS spectra for Ba 3*d* 10

Figure S9 Relaxed calculation model for Ba-TNS_OV_ 11

Figure S10 UV-vis DRS results 12

Figure S11 Standard curves for NH_4_^+^ 0.4 ppm to 2.0 ppm 13

Figure S12 Standard curves for NH_4_^+^ 1 ppm to 20 ppm 14

Figure S13 Ammonia synthesis efficiency with EG-OR15

Figure S14 Selectivity test results16

Figure S15 XRD patterns before and after long-term test 17

Figure S16 SEM images before and after long-term test 18

Figure S17 TEM images before and after long-term test 19

Figure S18 HAADF-STEM images before and after long-term test 20

Figure S19 Ammonia synthesis efficiency with OER21

Figure S20 Standard curves for HCOOH 22

Figure S21 *In-situ* DRIFTS spectra for combined NO_3_^-^ and HCHO 23

Figure S22 *In-situ* DRIFTS spectra for individual NO_3_^-^ 24

Figure S23 *In-situ* DRIFTS spectra for individual HCHO 25

Figure S24 Detected DMPO-^●^OH signals by EPR26

Figure S25 Detected TEMPO-e^-^ signals by EPR27

Figure S26 Primary pathways for NO_3_^-^ reduction on TNS_OV_ 28

Figure S27 Primary pathways for NO_3_^-^ reduction on Ba-TNS_OV_ 29

Figure S28 Side pathways for NO_3_^-^ reduction on Ba-TNS_OV_ 30

**Supplementary TablesS31**

Table S1 Ammonia synthesis efficiency comparison S31

Table S2 Formic acid synthesis efficiency comparison S33

Supplementary Figures


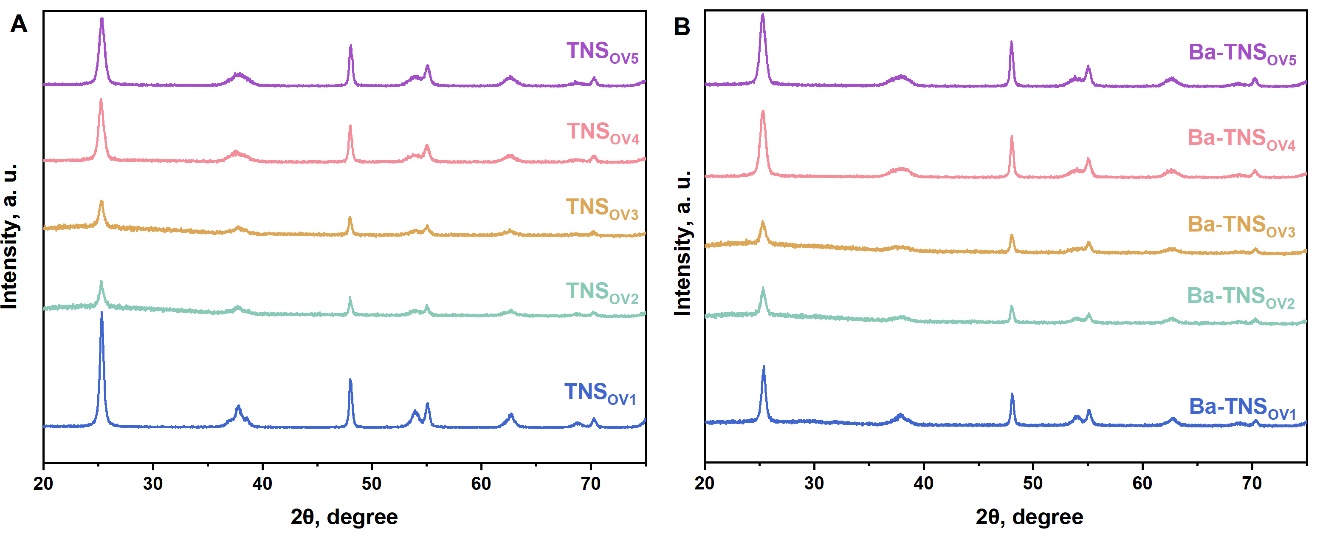


Fig. S1 XRD patterns for TNS_OV_ (A) and Ba-TNS_OV_ (B) samples.


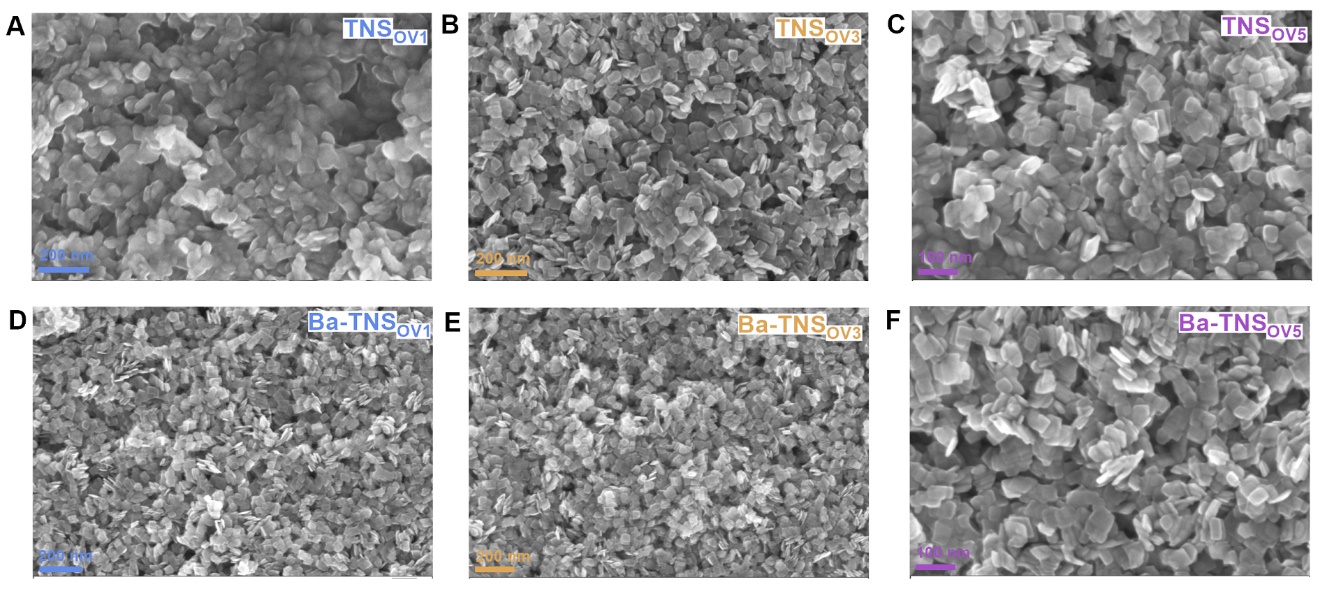
 Fig. S2 SEM images for TNS_OV_ (A-C) and Ba-TNS_OV_ (D-F) samples.


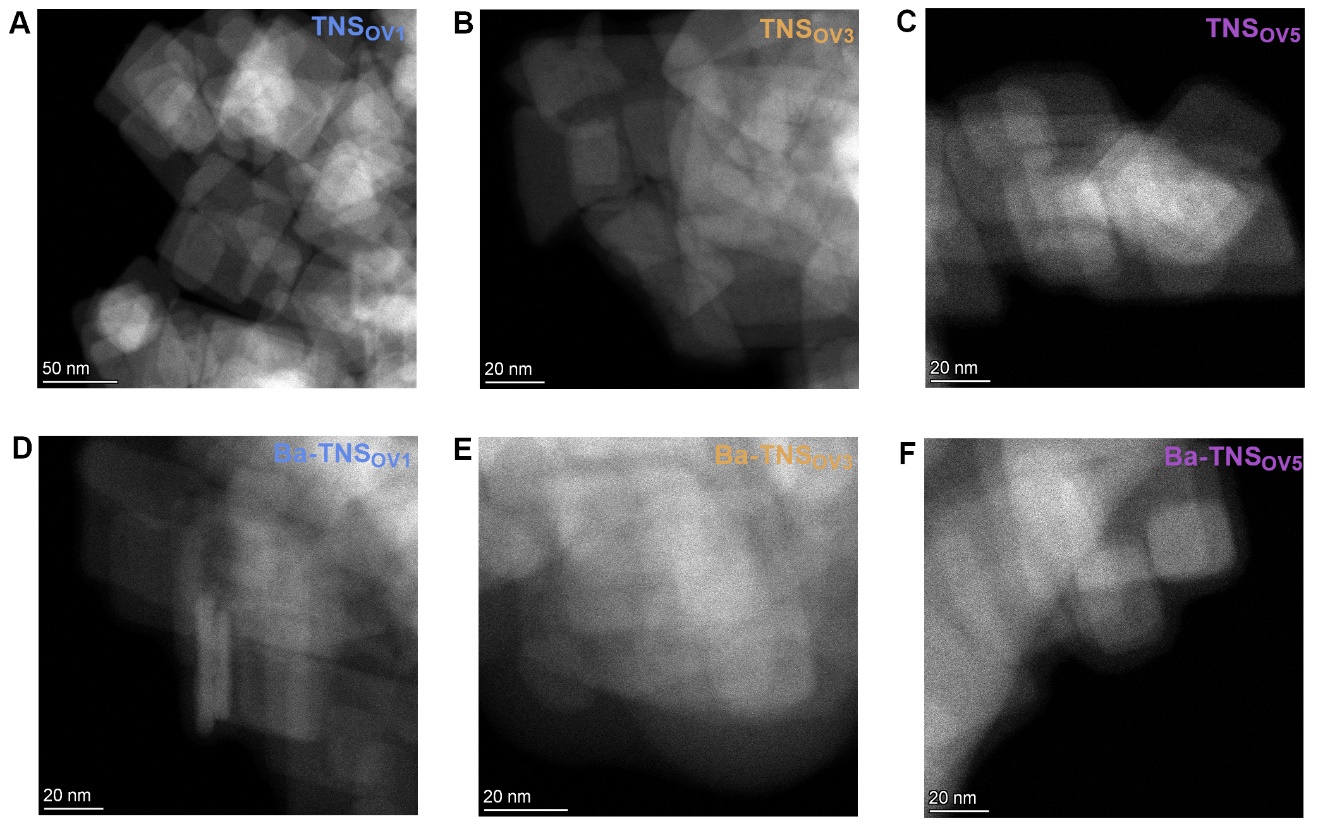


Fig. S3 TEM images for TNS_OV_ (A-C) and Ba-TNS_OV_ (D-F) samples.


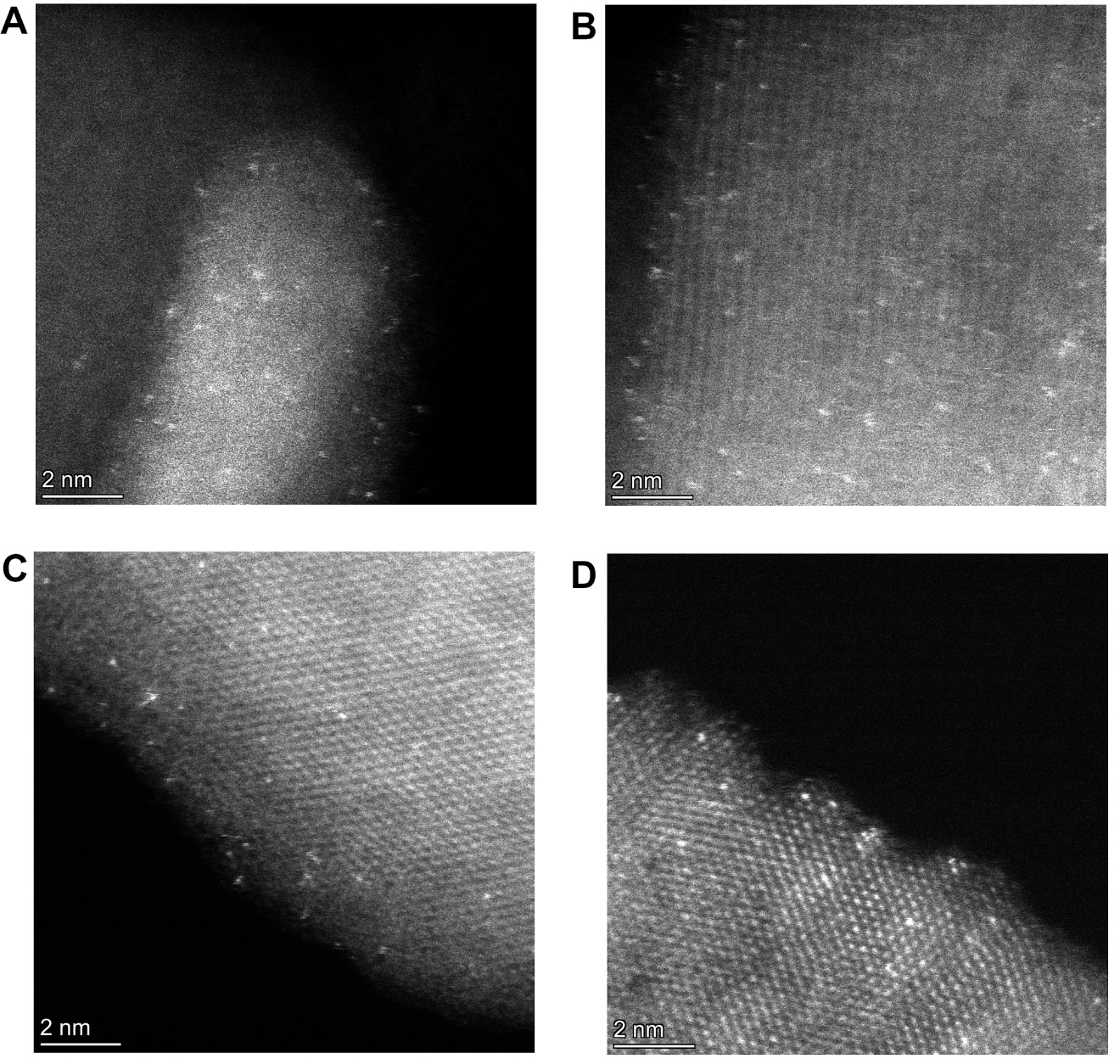


Fig. S4 HAADF-STEM images for Ba-TNS_OV2_ (A and B) and Ba-TNS_OV4_ (C and D).


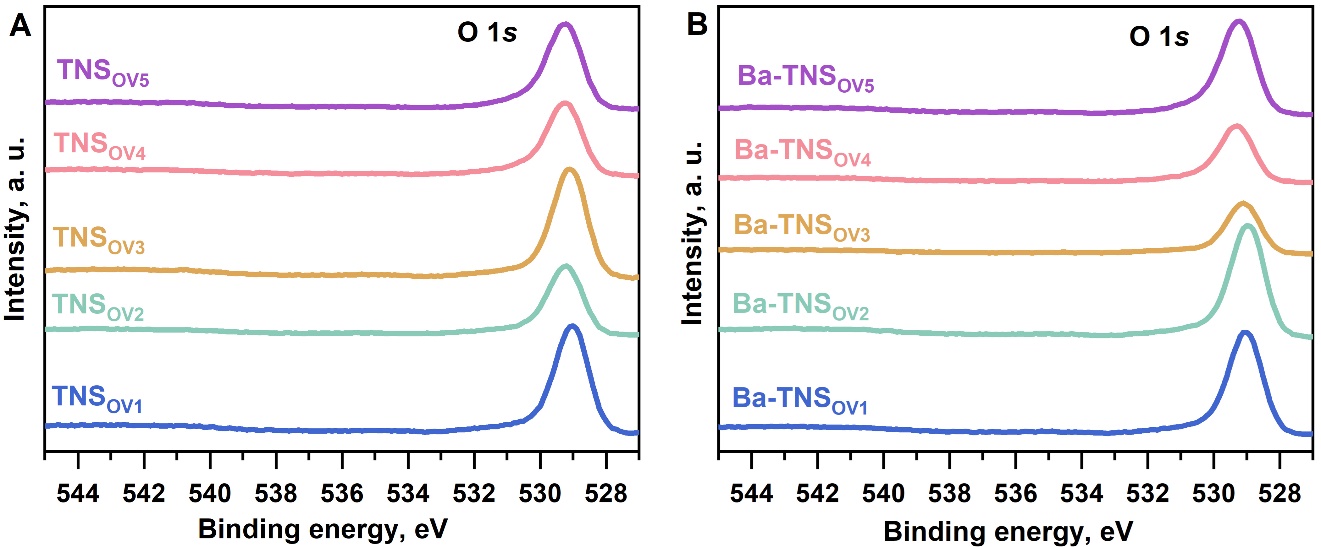


Fig. S5 High-resolved O 1*s* XPS spectra for TNS_OV_ (A) and Ba-TNS_OV_ (B) samples.


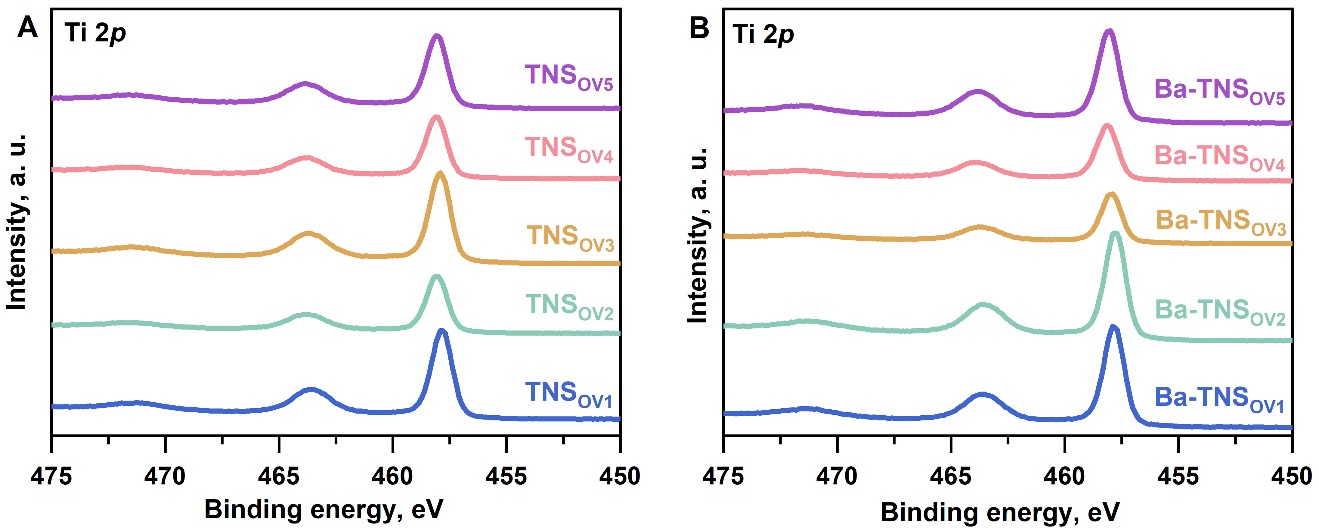


Fig. S6 High-resolved Ti 2*p* XPS spectra for TNS_OV_ (A) and Ba-TNS_OV_ (B) samples.


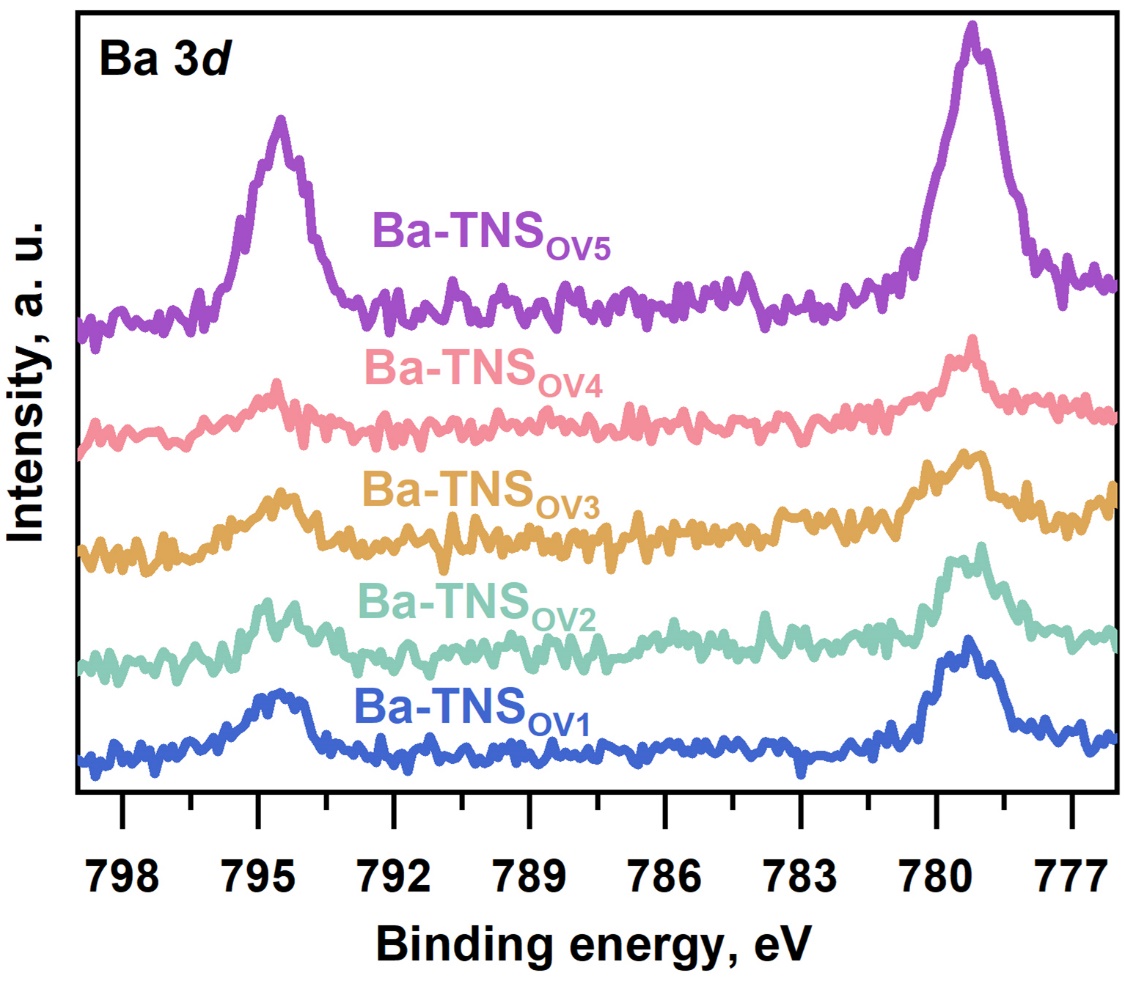


Fig. S7 High-resolved Ba 3*d* XPS spectra for Ba-TNS_OV_ samples.


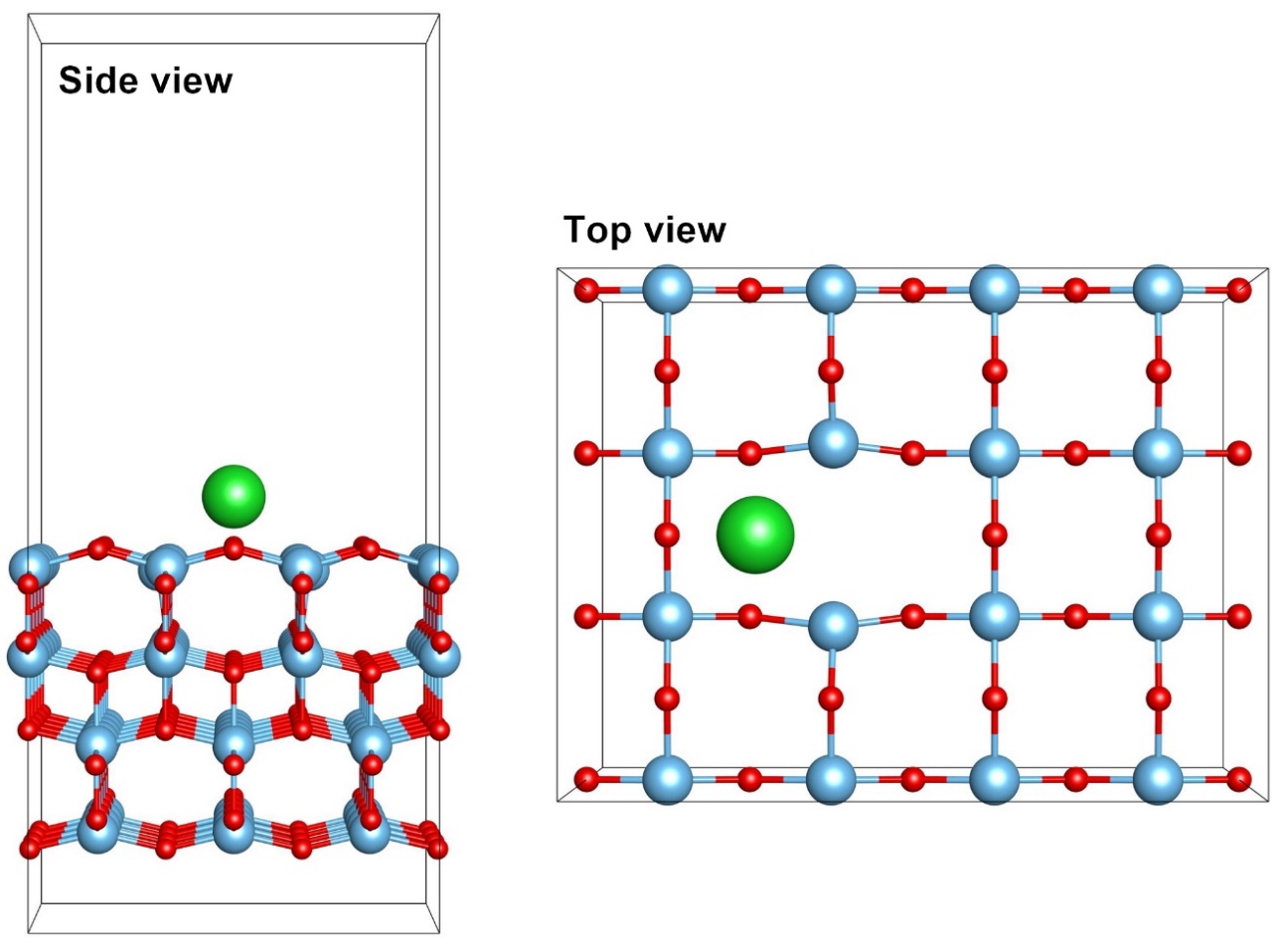


Fig. S8 Relaxed calculation model for Ba-TNS_OV_.


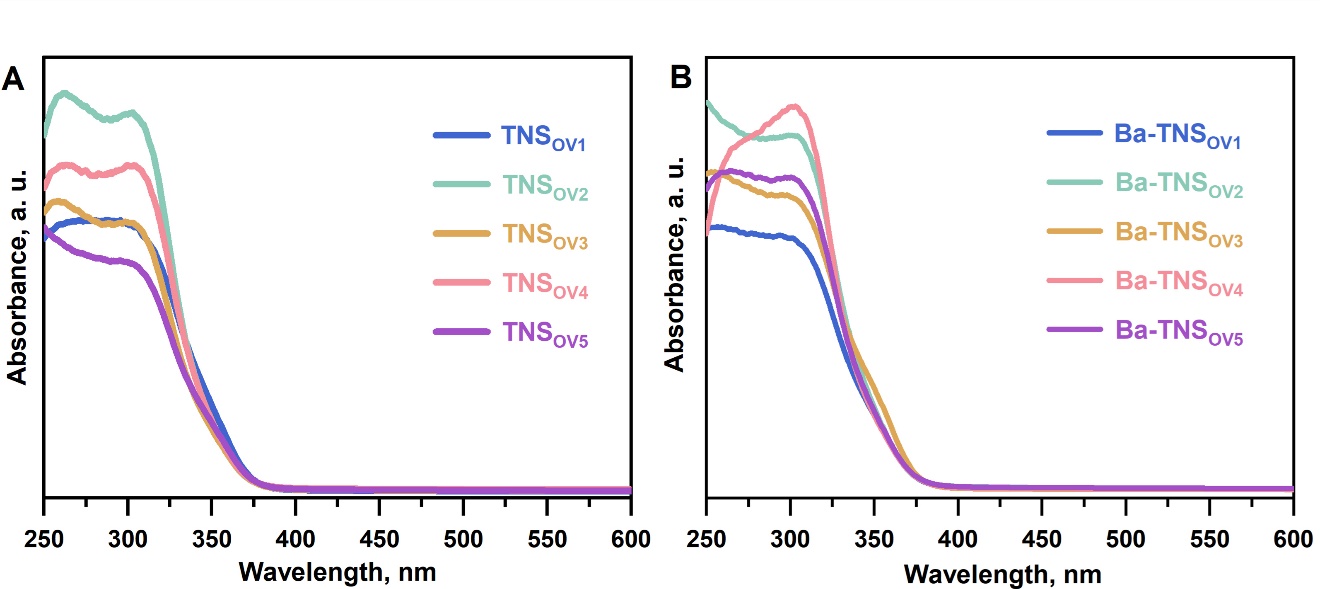


Fig. S9 UV-vis DRS results for TNS_OV_ (A) and Ba-TNS_OV_ samples.


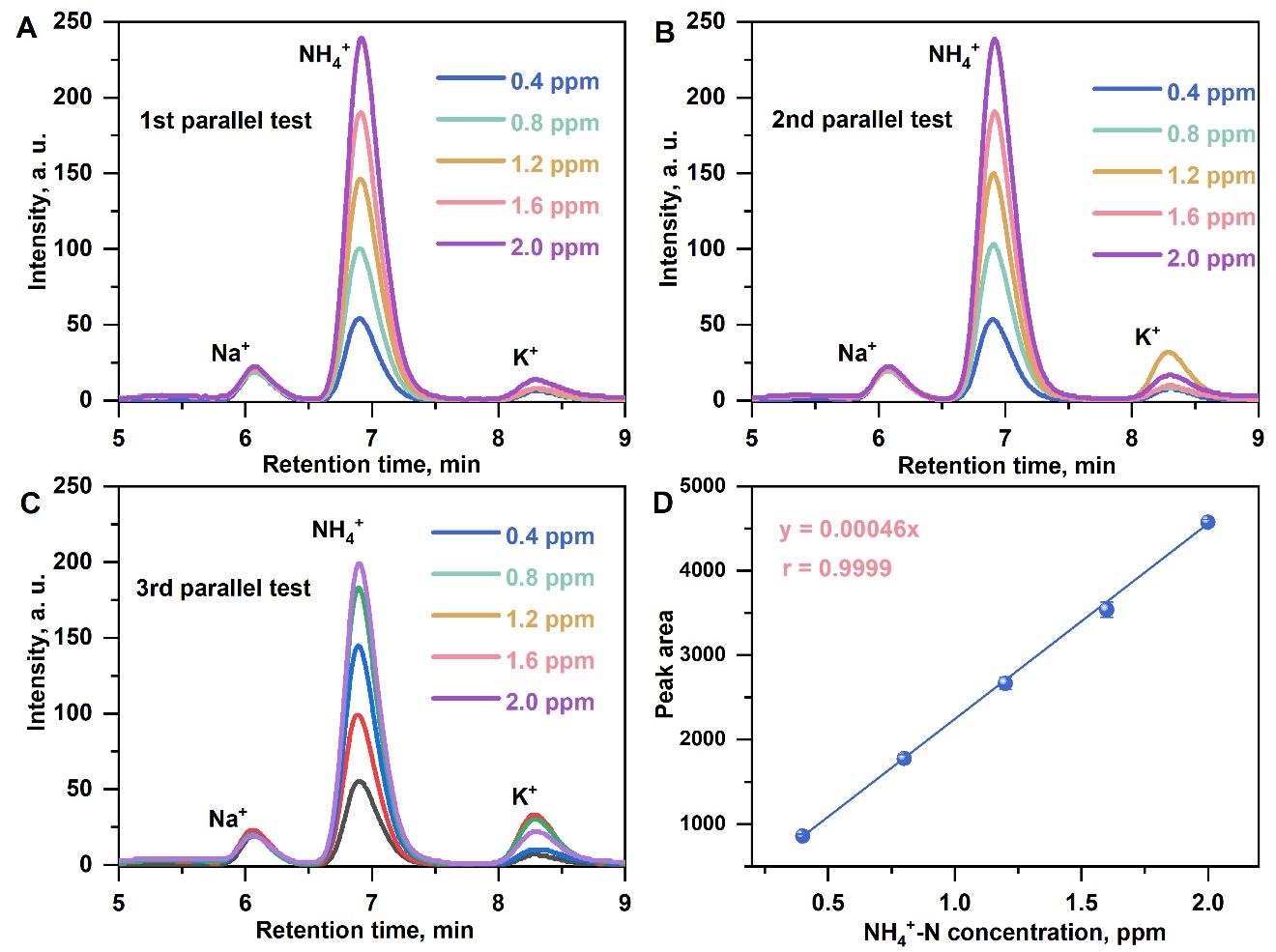


Fig. S10 Standard curves for NH_4_^+^ detection using ion chromatography from 0.4 ppm to 2.0 ppm.


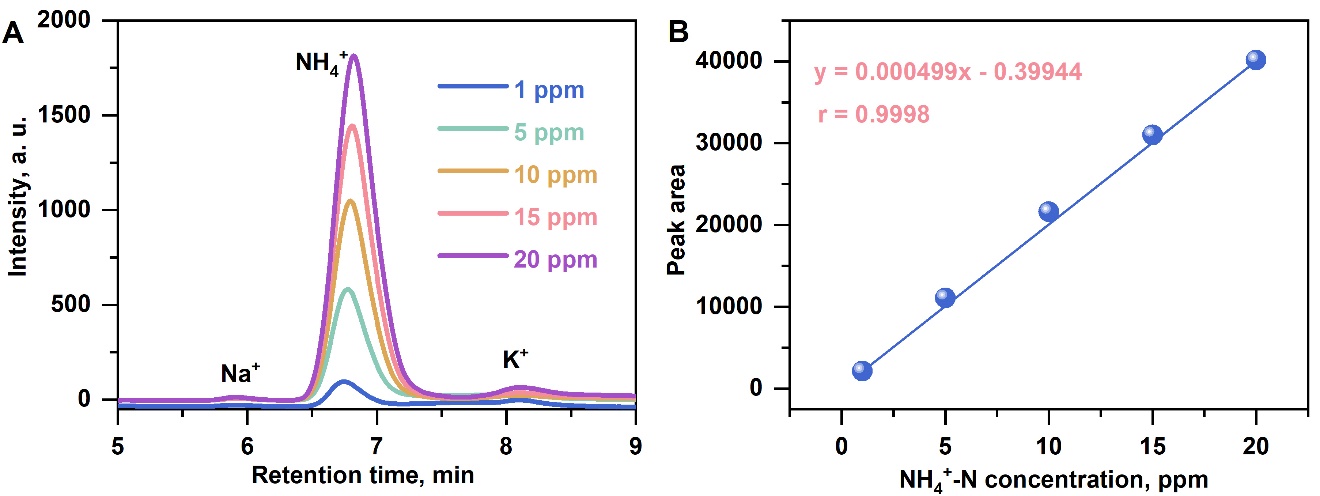


Fig. S11 Standard curves for NH_4_^+^ detection using ion chromatography from 1 ppm to 20 ppm.


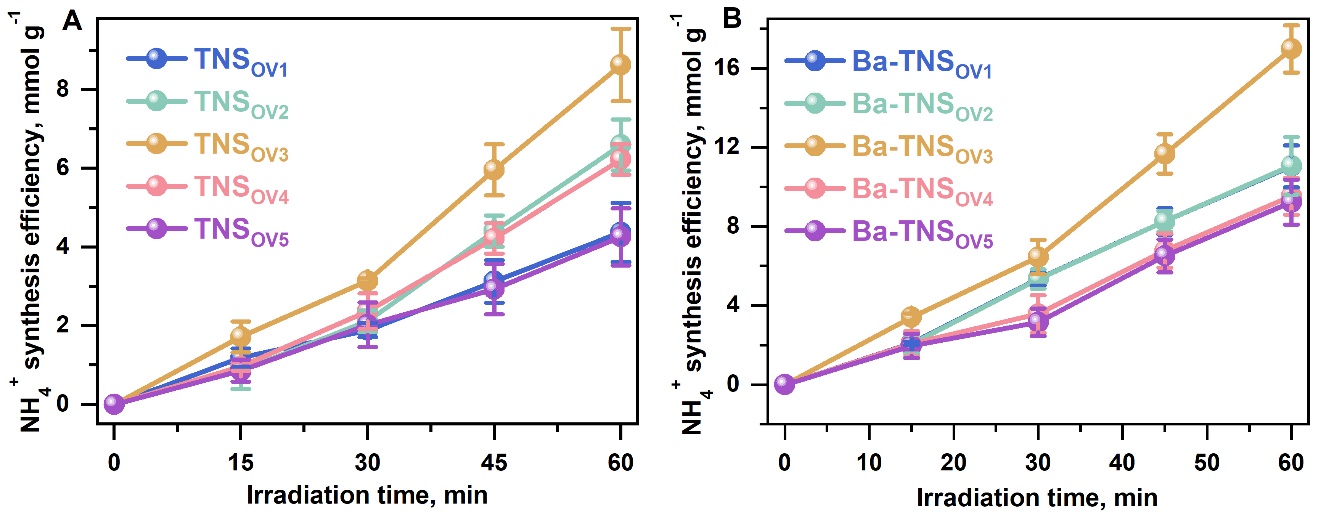


Fig. S12 Ammonia synthesis efficiency comparison within TNS_OV_ and Ba-TNS_OV_ samples.


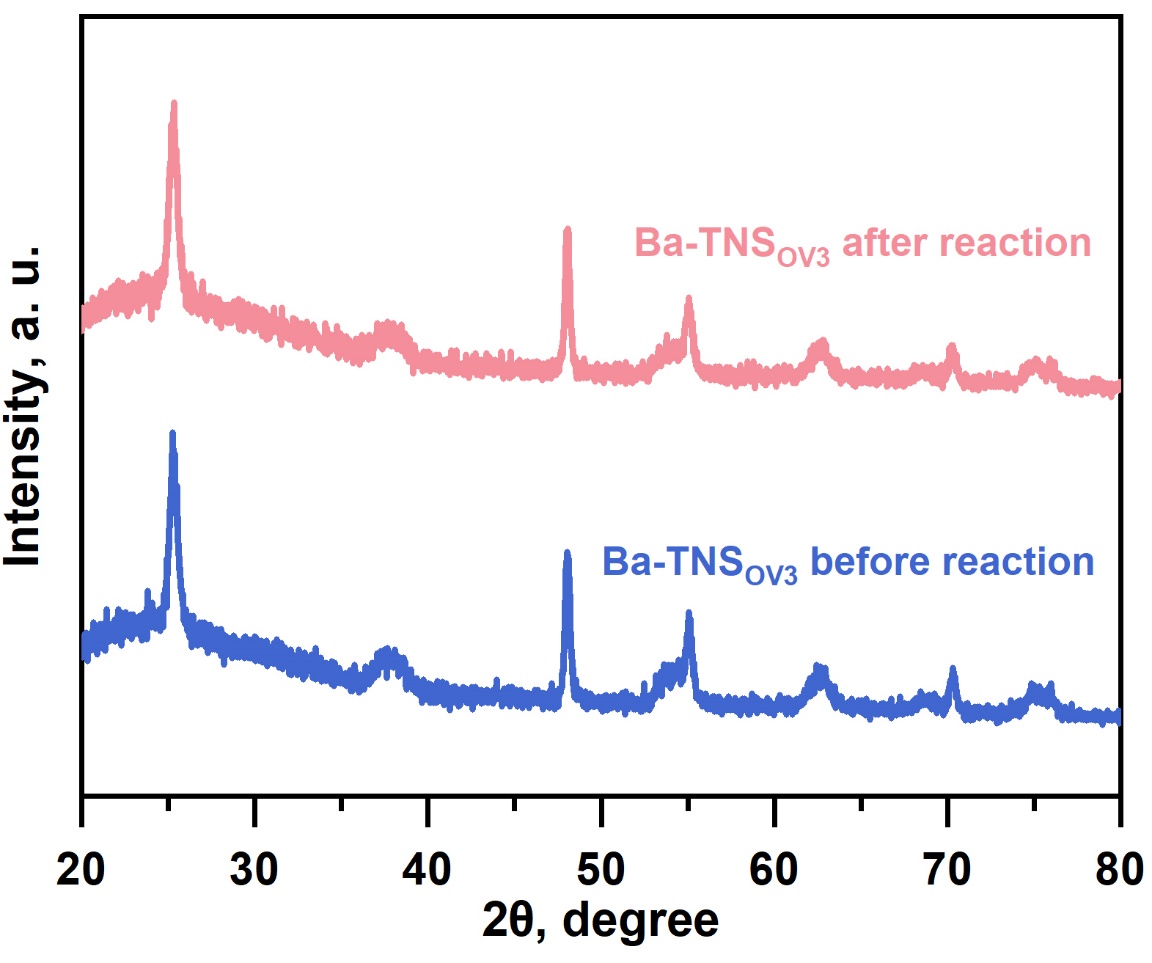


Fig. S13 XRD patterns for Ba-TNS_OV3_ before and after the long-term efficiency test.


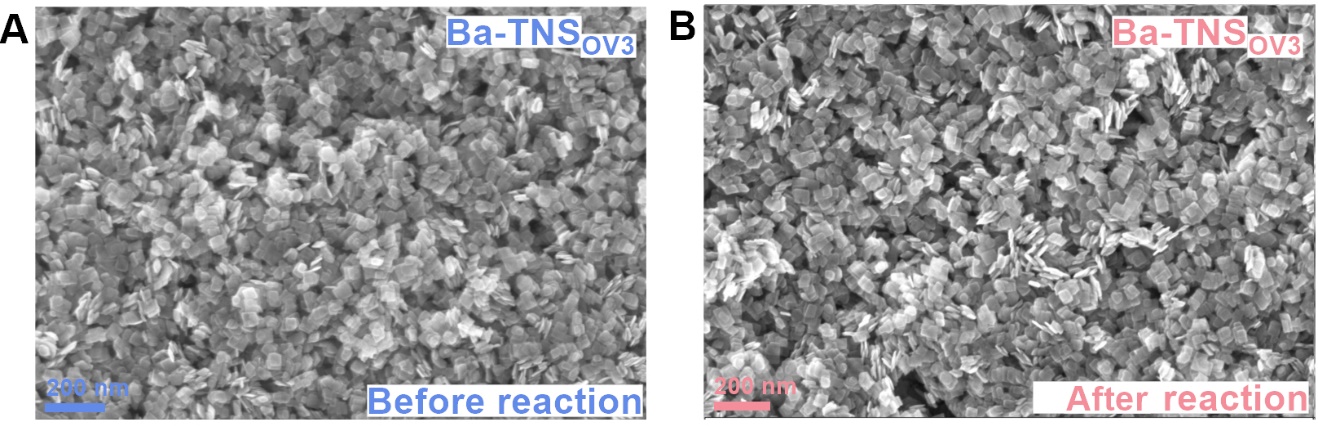


Fig. S14 SEM images for Ba-TNS_OV3_ before (A) and after (B) the long-term efficiency test.


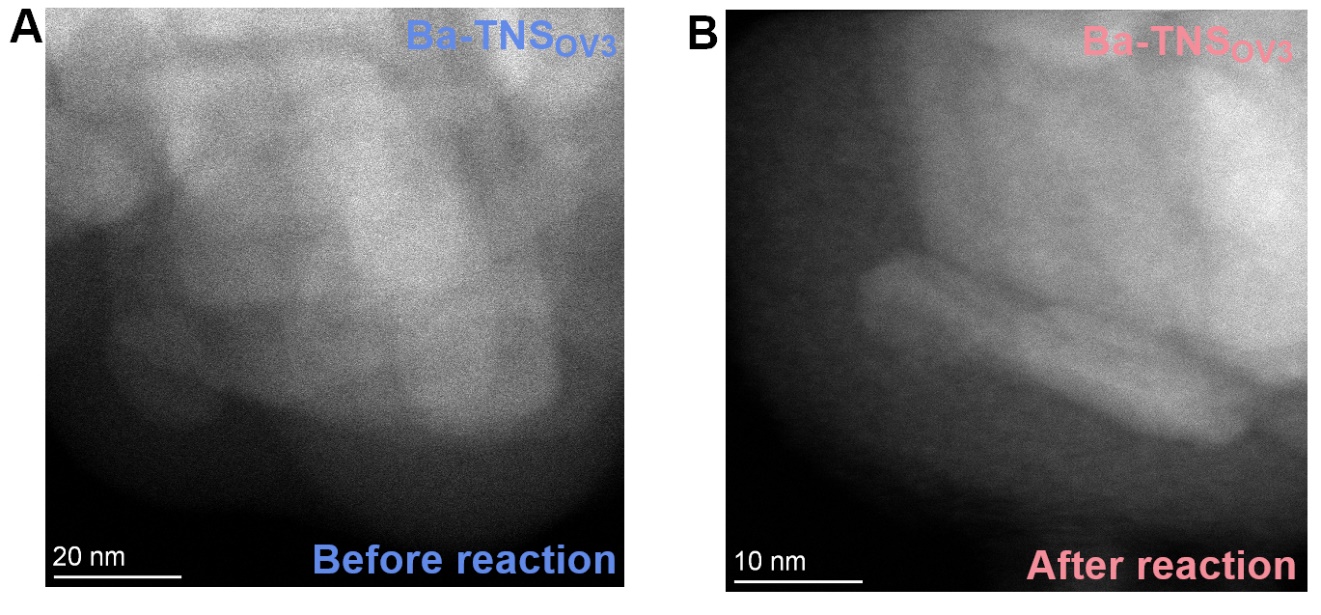


Fig. S15 TEM images for Ba-TNS_OV3_ before (A) and after (B) the long-term efficiency test.


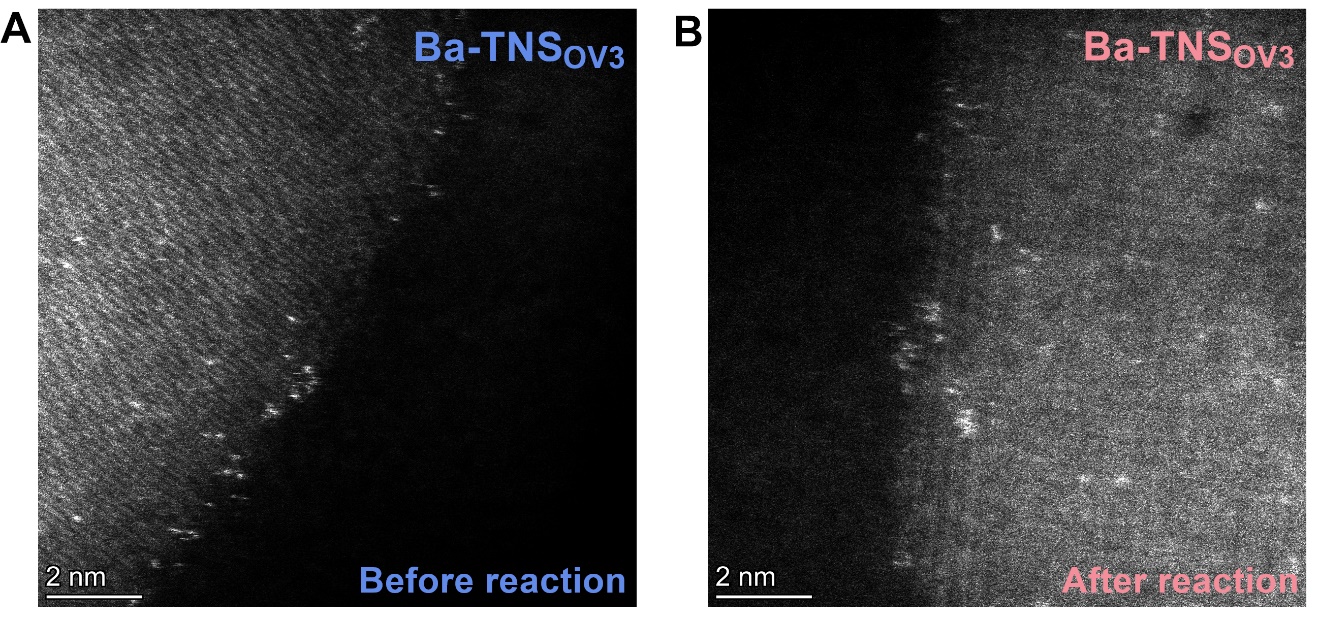


Fig. S16 HAADF-STEM images for Ba-TNS_OV3_ before (A) and after (B) the long-term efficiency test.


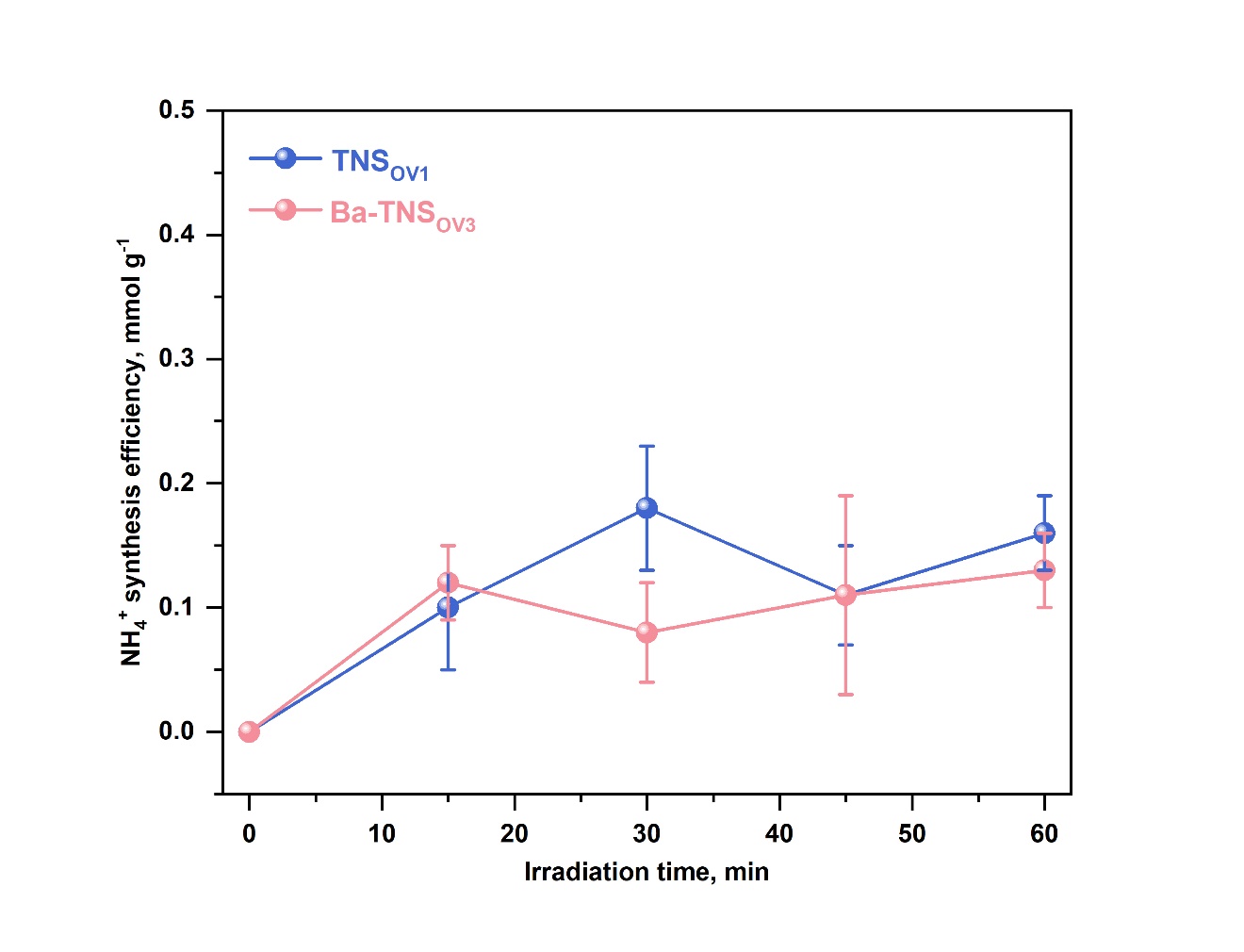


Fig. S17 Ammonia synthesis efficiency in the system of combined NO_3_^-^RR and OER.


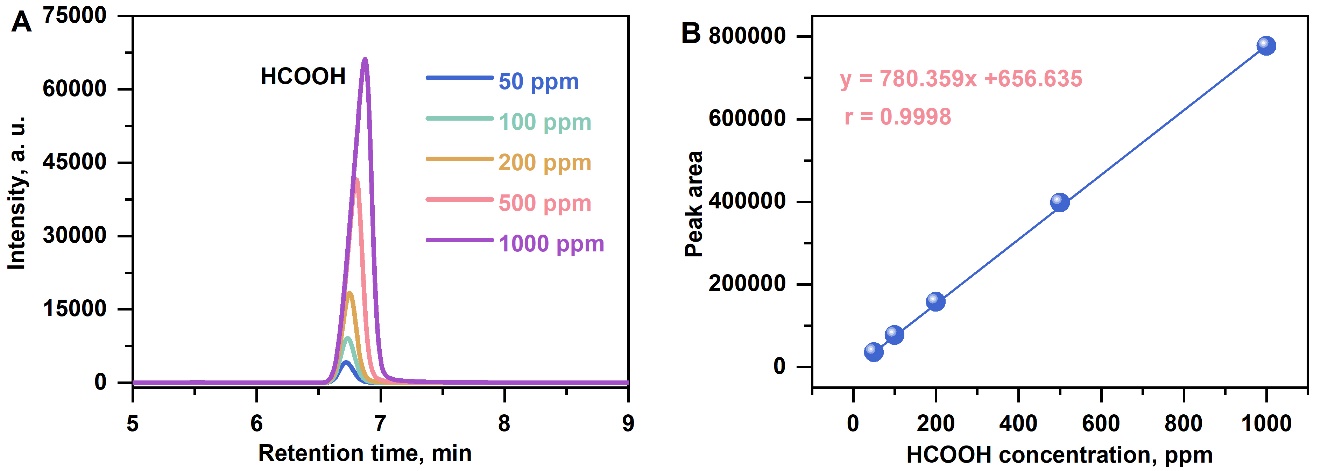


Fig. S18 Standard curves for HCOOH detection using ion chromatography from 50 ppm to 1000 ppm.


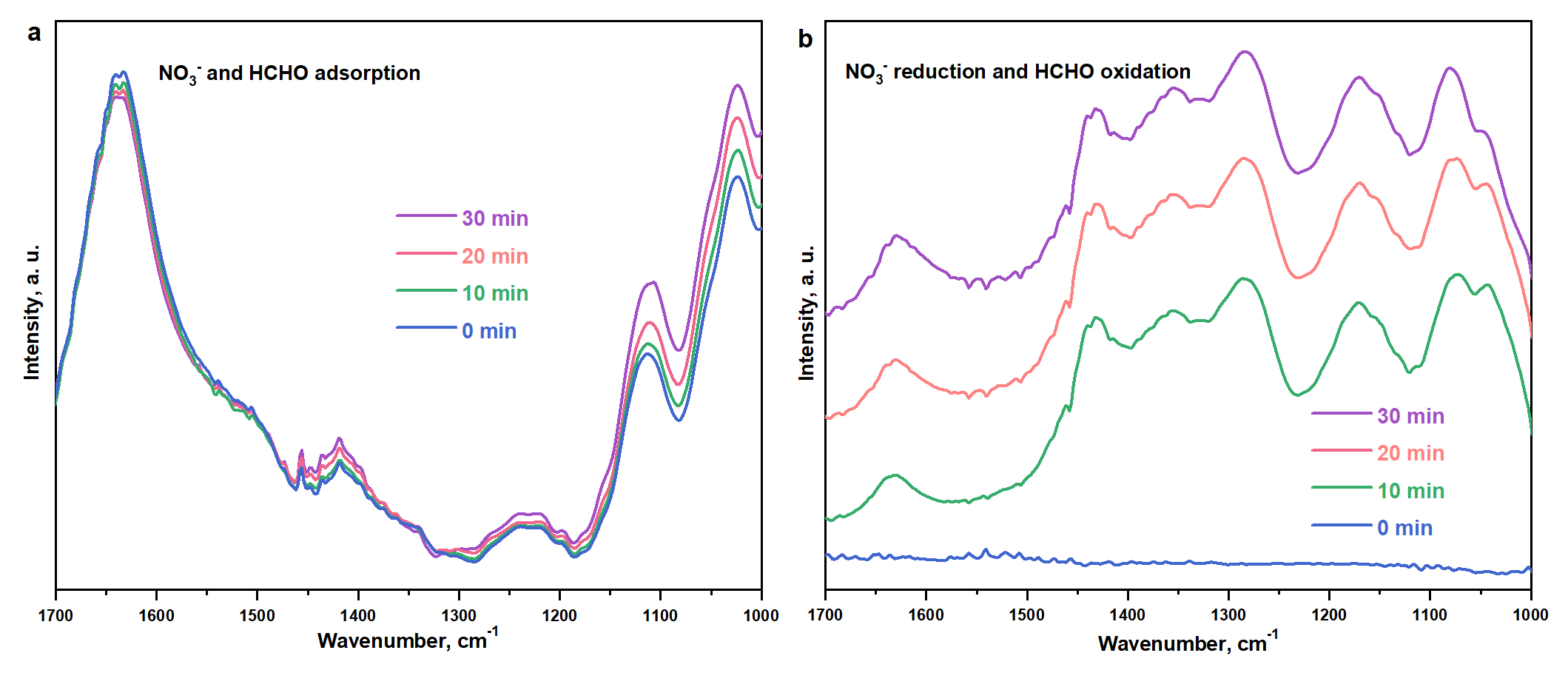


Fig. S19 *in-situ* DRIFTS spectra for combined NO_3_^-^ and HCHO adsorption (a) and NO_3_^-^ reduction and HCHO oxidation (b).


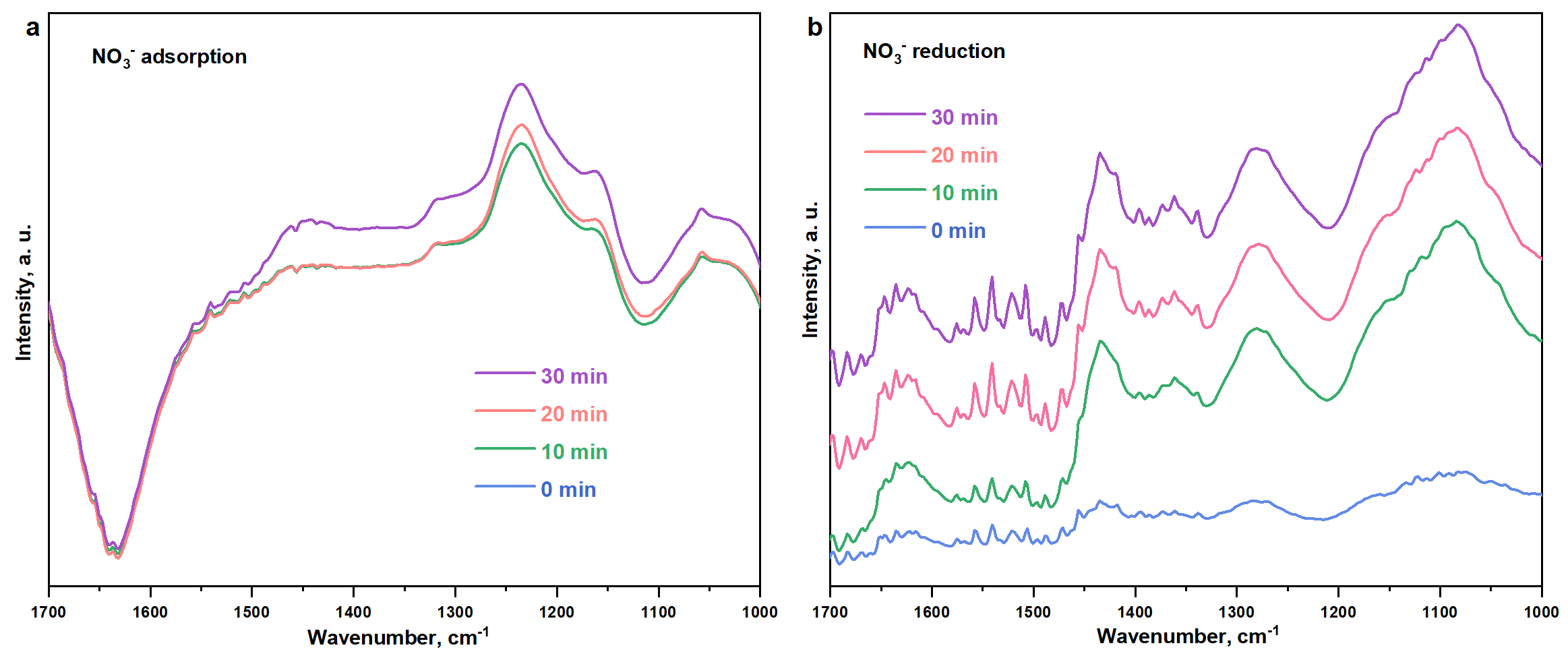


Fig. S20 *in-situ* DRIFTS spectra for individual NO_3_^-^ adsorption (a) and reduction (b).


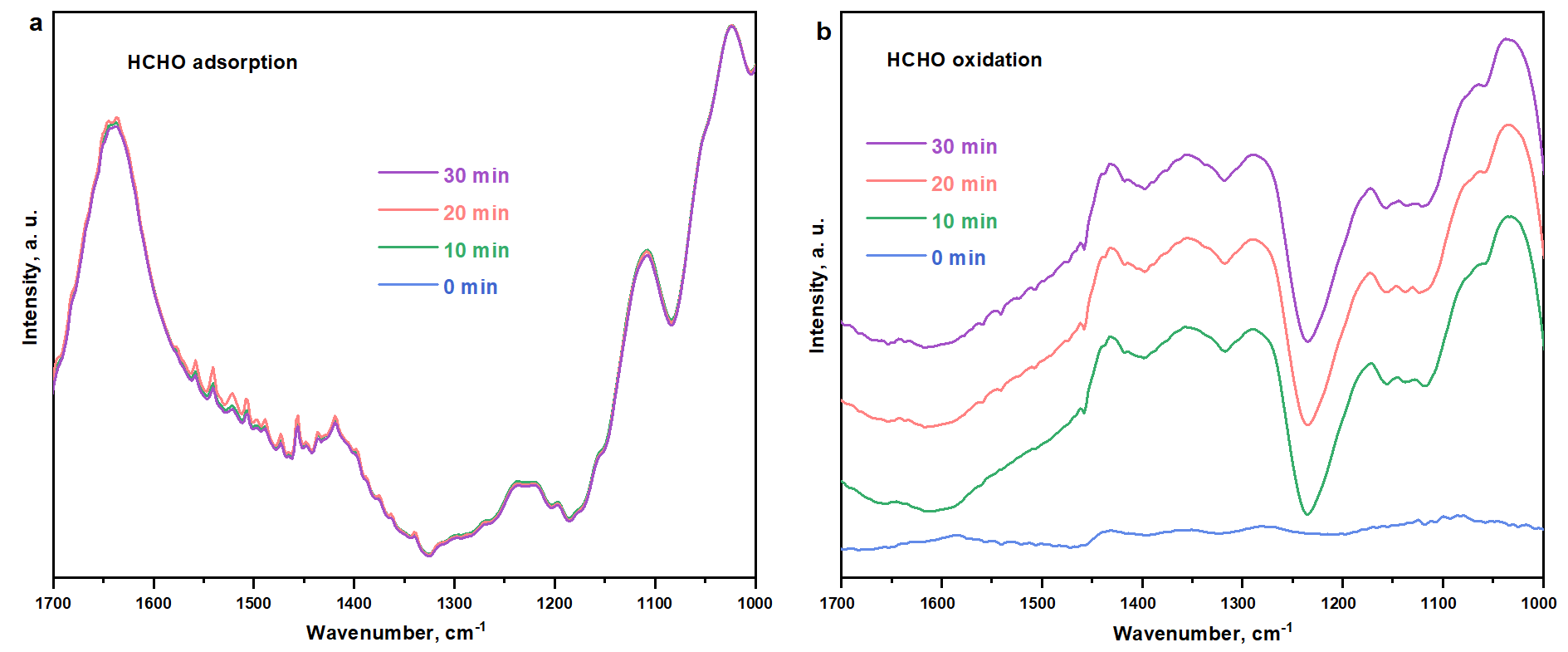


Fig. S21 *in-situ* DRIFTS spectra for individual HCHO adsorption (a) and oxidation (b).


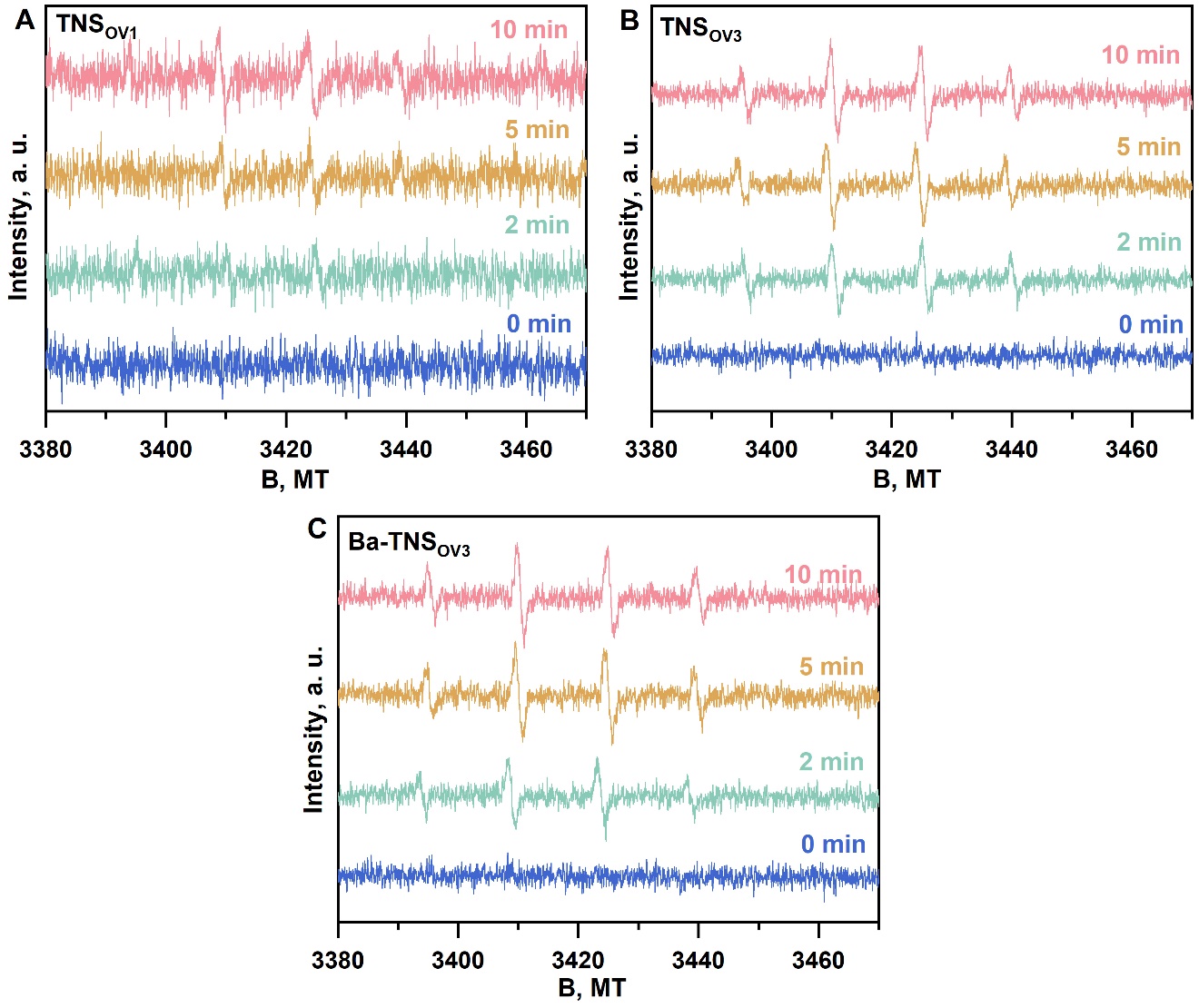


Fig. S22 Detected DMPO-^●^OH signals for TNS_OV1_ (A), TNS_OV3_ (B) and Ba-TNS_OV3_ (C).


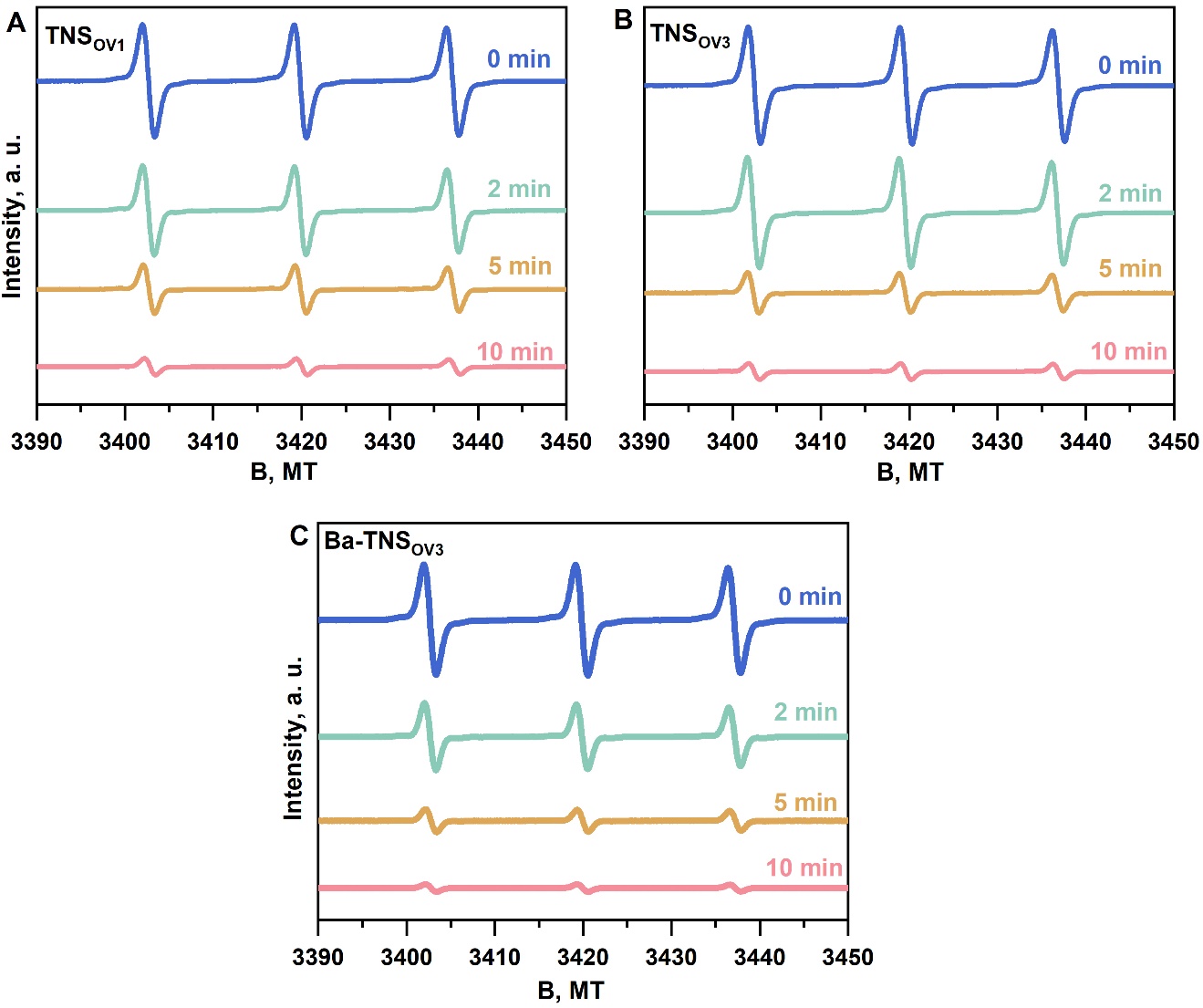


Fig. S23 Detected TEMPO-e^-^ signals for TNS_OV1_ (A), TNS_OV3_ (B) and Ba-TNS_OV3_ (C).


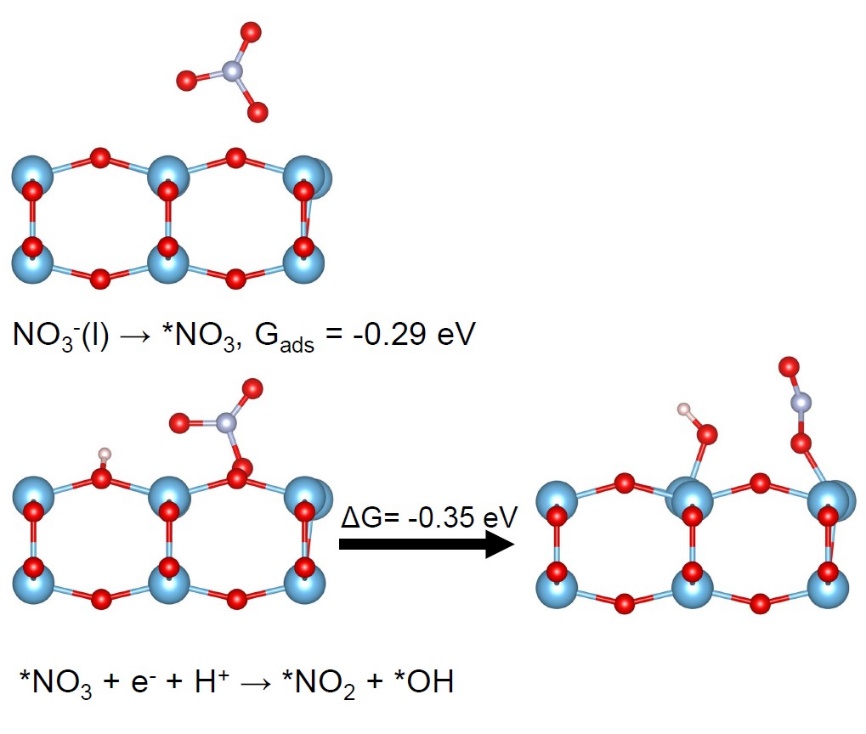


Fig. S24 Calculated primary pathways for NO_3_^-^ reduction on TNS_OV_ model.


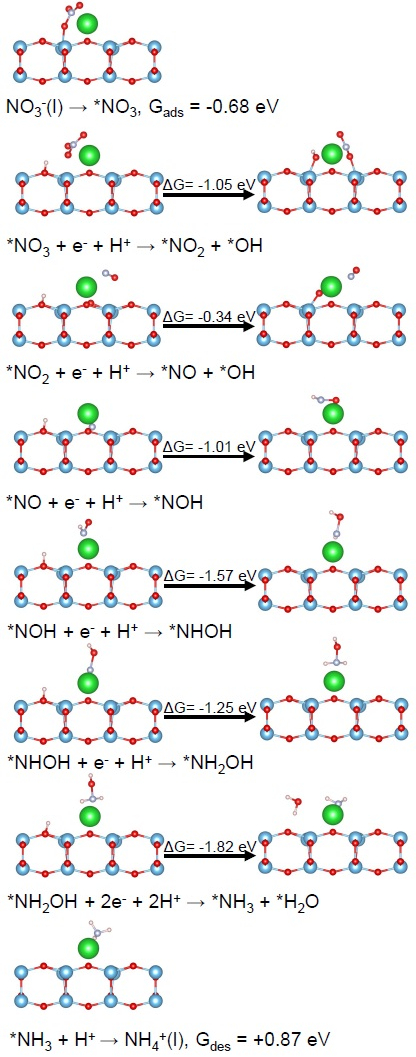


Fig. S25 Calculated primary pathways for NO_3_^-^ reduction on Ba-TNS_OV_ model.


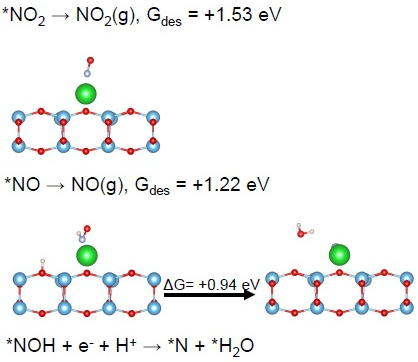


Fig. S26 Calculated side pathways for NO_3_^-^ reduction on Ba-TNS_OV_ model.

**Supplementary Tables**

Table S1 Ammonia synthesis efficiency comparison between this work and the other photocatalysis routes.

| **Ammonia**  **synthesis route** | **Catalyst** | **ammonia**  **synthesis rate**  **(mmol g_cat_^-1^ h^-1^)** | **Reference** |
| --- | --- | --- | --- |
| Photocatalytic NO_3_^-^RR | BaO_NCs_@TiO_2_ | 15.80 | *Nat. Commun.*,  2022, 13, 1098. |
| Photocatalytic NO_3_^-^RR | JRC-TIO-6 | 0.016 | *ACS Catal.*,  2017, *7*, 3713-3720. |
| Photocatalytic NO_3_^-^RR | Ag_2_O/P25 | 0.45 | *Appl. Catal. B: Environ.*,  2015, *176-177*, 53-61. |
| Photocatalytic NO_3_^-^RR | Au/TiO_2_ | 0.69 | *Catal. Today*,  2012, *181*, 171-176. |
| Photocatalytic NO_3_^-^RR | BiO-160 | 0.46 | *J. Cleaner Prod.*,  2022, *331*, 129975. |
| Photocatalytic N_2_RR | OVs-Bi_5_O_7_Br | 12.72 | *J. Am. Chem. Soc.*,  2020, *142*, 12430-12439. |
| Photocatalytic N_2_RR | K^+^@g-C_3_N_4_ | 3.42 | *Angew. Chem. Int. Ed.*,  2019, *58*, 16644-16650. |
| Photocatalytic N_2_RR | ZnCr-LDH | 0.03 | *Adv. Energy Mater.*,  2020, *10*, 2002199. |
| Photocatalytic N_2_RR | OV-TiO_2_ | 0.08 | *Adv. Mater.*,  2019, *31*, 1806482. |
| Photocatalytic N_2_RR | Au@UiO-66 | 0.81 | *J. Am. Chem. Soc.*,  2021, *143*, 5727-5736. |
| Photocatalytic N_2_RR | Fe-TiO_2_ | 0.06 | *Angew. Chem. Int. Ed.*,  2021, *60*, 16085-16092*.* |
| Photocatalytic N_2_RR | Al-PMOF (Fe) | 0.007 | *ACS Nano*,  2021, *15*, 9670-9678. |
| **Photoredox**  **NO_3_^-^RR with EG-OR** | **Ba-TNS_OV3_** | **16.98** | ***This work*** |
| **Photoredox**  **NO_3_^-^RR with HCHO-OR** | **Ba-TNS_OV3_** | **31.99** | ***This work*** |

Table S2 Formic acid (FA) synthesis efficiency comparison between this work and the other synthesis routes.

| **FA**  **synthesis route** | **Catalyst** | **ammonia**  **synthesis rate**  **(mmol g_cat_^-1^ h^-1^)** | **Reference** |
| --- | --- | --- | --- |
| Thermocatalytic  methanol oxidation  *T* = 100℃, *P* = 20 Bar | Au/TiO_2_ | 0.46 | *Ind. Eng. Chem. Res.*,  2017, 56, 11028-11033. |
| Thermocatalytic  glycerol oxidation  *T* = 160℃, *P* = 5 Bar | Ru(OH)_4_/r-GO | 1.52 | *Appl. Catal. B: Environ.*,  2014, 154-155, 167-273. |
| Thermocatalytic  methane oxidation  *T* = 50℃, *P* = 30 Bar | Z20M | 7.66 | *Fuel*, 2021, 305, 121624. |
| Thermocatalytic  methane oxidation  *T* = 50℃, *P* = 30 Bar | UiO-66(2.5TFA)-Fe | 3.11 | *Angew. Chem. Int. Ed.*,  2021, 60, 5811-5815. |
| Thermocatalytic  methane oxidation  *T* = 70℃, *P* = 3 Bar | AuPd@ZSM-5-C | 0.59 | *Science*, 2020, 367, 193-197. |
| Thermocatalytic  methane oxidation  *T* = 50℃, *P* = 3 Bar | Fe/ZSM-5 | 3.89 | *Chem. Sci.*,  2021, 12, 3152-3160. |
| Photocatalytic  methanol oxidation | Co@PNC | 0.06 | *Chem. Commun.*,  2020, 56, 4664-4667. |
| Photocatalytic  glucose oxidation | TiO_2_ | 2.95 | *ACS Sustainable Chem. Eng.*,  2017, 5, 6377-6381. |
| Photocatalytic  glucose oxidation | Bi_2_WO_6_/CoPz | 0.27 | *Appl. Catal. A: Gen.*,  2021, 623, 118265. |
| Photocatalytic  glucose oxidation | TiO_2_ (P25) | 0.05 | *Green Chem.*,  2019, 21, 5019-5029. |
| Photocatalytic  glucose oxidation | H-ZSM-5/FePz(SBu)_8_ | 0.03 | *J. Catal.*, 2019, 374, 297-305. |
| Photocatalytic  glycerol oxidation | Au-PTA/MSN | 3.32 | *ACS Sustainable Chem. Eng*., 2021, 9, 3571-3579. |
| **Photoredox**  **HCHO-OR with NO_3_^-^RR** | **Ba-TNS_OV3_** | **54.11** | ***This work*** |
